# Modelling Atherosclerotic Plaque Cap Mechanics: Microcalcifications Reduce Mechanical Properties in Mesenchymal Stromal Cell‐Based Model

**DOI:** 10.1002/adbi.202500106

**Published:** 2025-07-09

**Authors:** Imke L. Jansen, Deniz Şahin, Frank J.H. Gijsen, Eric Farrell, Kim van der Heiden

**Affiliations:** ^1^ Department of Biomedical Engineering Thorax Center Erasmus MC University Medical Center Rotterdam Rotterdam 3015 GD The Netherlands; ^2^ Department of Biomechanical Engineering Delft University of Technology Delft 2628 CD The Netherlands; ^3^ Department of Oral and Maxillofacial Surgery Erasmus MC University Medical Center Rotterdam Rotterdam 3015 GD The Netherlands

**Keywords:** atherosclerosis, cardiovascular system, cell cultures, mechanical testing, multiphoton microscopy, tissue engineering

## Abstract

Rupture of atherosclerotic plaque caps is the cause of many disabling or lethal cardiovascular events, such as stroke and myocardial infarction. Microcalcifications (<50 µm) have been shown, in computational models, to affect the biomechanical stability of the cap. The current study aims to develop a tissue‐engineered model of the atherosclerotic fibrous cap with microcalcifications produced by mesenchymal stromal cells (MSCs). Human MSCs are seeded in fibrin gels and cultured for 2 weeks in medium supplemented with TGF‐β1 to induce smooth muscle cell differentiation and collagenous matrix formation. Afterward, mineralizing medium stimulates microcalcification formation for an additional 4 weeks. Tissue‐engineered structures are imaged after culture with second harmonic generation microscopy with a hydroxyapatite probe, showing collagenous matrix with microcalcifications. Mechanical characterization shows the effect of microcalcifications on global tissue mechanics, as the ultimate stress at rupture of the tissue is significantly lower compared to control tissues. The amount of calcification, determined by histological analysis, is correlated to the decrease in ultimate tensile stress, with a higher amount of microcalcification resulting in weakened mechanical properties. The developed tissue‐engineered plaque cap model with biologically formed collagenous matrix and microcalcifications offers valuable insight into the impact of microcalcifications on biomechanical stability.

## Graphical abstract

This study develops a tissue‐engineered model of the atherosclerotic cap using human mesenchymal stromal cells (MSCs). After 2 weeks of culture to produce a collagenous matrix, a mineralizing medium induces microcalcifications over 4 weeks. These constructs, imaged with second harmonic generation microscopy, lead to reduced ultimate stress at rupture, highlighting the model's relevance for studying biomechanical stability in cardiovascular events.

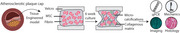

## 1 Introduction

Rupture of the atherosclerotic plaque cap is the cause of many disabling or lethal cardiovascular events, such as stroke and myocardial infarction.^[^ ^1^, ^2^, ^3^ ^]^ Previously, fibrous cap thickness was considered the main determinant associated with plaque rupture risk, but the presence of other cap components, such as microcalcifications (<50 µm^[^ ^1^ ^]^) and macrophages, have also been shown to affect the biomechanical stability of the cap.^[^ ^1^, ^4^, ^5^, ^6^ ^]^ The microcalcifications are composed of hydroxyapatite^[^ ^7^, ^8^, ^9^, ^10^ ^]^ and it has been shown in both numerical^[^ ^11^, ^12^, ^13^, ^14^, ^15^, ^16^, ^17^ ^]^ and experimental^[^ ^18^, ^19^ ^]^ studies that they can increase the risk of cap rupture through the promotion of high local stress accumulations within the cap.

So far, multiple mechanisms that govern the formation of microcalcifications have been put forward. One potential pathway is the osteogenic trans‐differentiation of vascular smooth muscle cells (VSMCs) into osteoblast‐like cells. Multiple lines of evidence have shown that atherosclerotic calcification shares features with bone formation. Furthermore, bone‐related proteins (among others RUNX2 and ALP) have been observed in atherosclerotic lesions.^[^ ^20^, ^21^, ^22^ ^]^ The similarities with bone formation has caused the speculation to arise that mesenchymal stromal cells (MSCs) might be involved in the process of vascular calcification.^[^ ^23^, ^24^ ^]^ It has been shown that MSCs can migrate into the media, differentiate into VSMCs and later turn into osteoblast‐like cells.^[^ ^23^ ^]^

To provide further insight into the pathway by which microcalcifications form, their role in cap mechanics and their interplay with other cap components, systematic experimental studies are needed. *Ex vivo* material is often obtained from ruptured human plaques. However, this material is already damaged and has a very heterogenous composition. As an alternative, animal models are frequently used, but are limited due to ethics and the inability to mimic the human mechanical plaque environment.^[^ ^25^ ^]^ Clinical studies currently have difficulty in correlating the presence of microcalcifications to clinical events. This is mainly due to the heterogenous composition of plaques, causing confounding factors.^[^ ^26^ ^]^ Additionally, due to limited resolution of current clinical imaging modalities, many clinical studies focus on macrocalcification scoring, without capturing the role of microcalcifications.^[^ ^1^ ^]^ Therefore, 3D in vitro models with relevant biological components, suitable for mechanical testing are needed.

In order to tissue engineer an atherosclerotic cap comprised of a collagenous matrix and microcalcifications, multiple cell types might be used. VSMCs are physiologically relevant and calcification can be induced in vitro,^[^ ^27^ ^]^ as was shown with bovine^[^ ^28^ ^]^ and mouse^[^ ^29^, ^30^ ^]^ cells. However, if human cells are to be used, VSMCs need to be isolated during an invasive treatment and furthermore have a low proliferation capacity.^[^ ^31^, ^32^ ^]^ An alternative option is to use MSCs as they are easily accessible from various anatomical sources, such as bone marrow and adipose tissue.^[^ ^31^, ^33^, ^34^ ^]^ Furthermore, they are highly proliferative and can differentiate into various cell types, such as SMCs.^[^ ^32^, ^35^, ^36^, ^37^, ^38^, ^39^ ^]^ Various culture conditions have been proposed to induce such differentiation, such as the addition of the growth factor TGF‐β1,^[^ ^32^, ^35^, ^37^, ^38^, ^39^, ^40^ ^]^ mechanical stress^[^ ^41^, ^42^ ^]^ and cell‐surface interactions.^[^ ^36^, ^43^ ^]^ These studies were performed in monolayer cultures, while for a tissue engineered cap a 3D environment would be a prerequisite. Furthermore, to date, it has not been investigated whether SMCs differentiated from MSCs are capable of calcifying in an in vitro environment. In this study we create a biologically relevant tissue engineered (TE) construct of the atherosclerotic plaque cap with collagenous matrix and microcalcifications, following the pathway of vascular calcification using MSCs. We show that MSCs can differentiate into a SMC‐like phenotype in a 3D environment, after which they can be stimulated to calcify and form microcalcifications. Uniaxial tensile testing was used to evaluate the mechanical role of microcalcifications in these TE plaque caps, showing that calcified tissues have reduced ultimate stress and stiffness and consequently can influence plaque rupture.

## 2 Results

### 2.1 Medium Selection for Differentiation to SMC‐Like Phenotype in Monolayer

To select the medium for differentiation from an MSC to a SMC‐phenotype, different base media and supplements were first assessed in monolayer culture. qPCR showed an increase in gene expression of αSMA and Calponin for all medium types (Figure S1A, Supporting Information). Brightfield microscopy showed decreased cellular proliferation in the groups supplemented with 1% of FBS compared to 10% FBS (Figure S1B, Supporting Information). After an additional culture of 15 days in osteogenic medium, cells were stained with Alizarin Red. The groups which started with αMEM or LG‐DMEM supplemented with 10% FBS during initial differentiation showed the most calcification compared to the groups that differentiated in HG‐DMEM (Figure S1C, Supporting Information). Since cells differentiated with αMEM and 10% FBS showed microcalcifications with increased diameter, compared to those differentiated in LG‐DMEM, αMEM was chosen for as the culture medium of the differentiation from MSC to SMC phenotype in the 3D TE constructs.

### 2.2 TGF‐β1 Supplementation Causes Collagen Formation and Differentiation to SMC‐Like Phenotype

**Figure 1:**
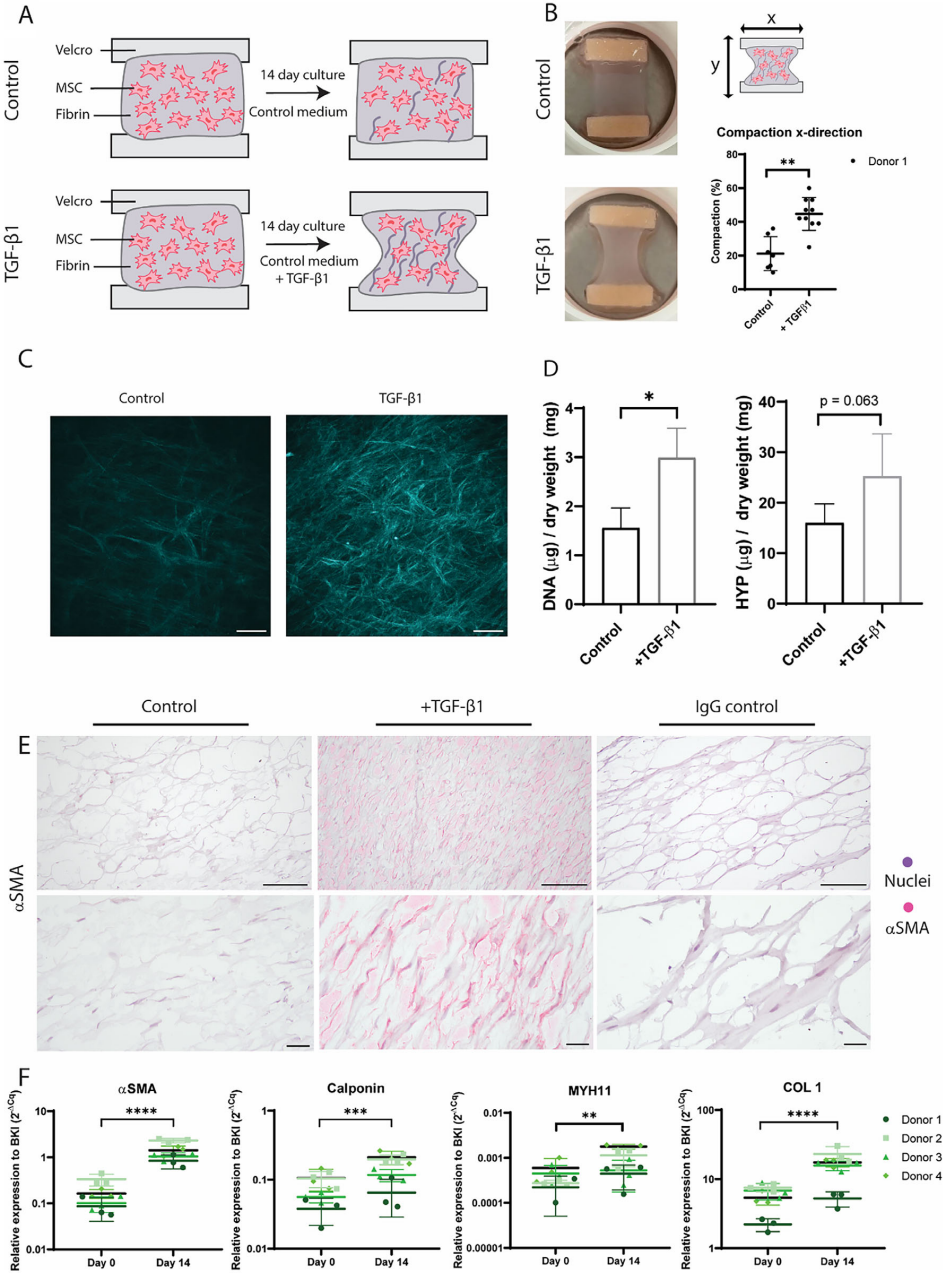
Creation of TE‐construct with collagenous matrix from MSCs to SMC‐like phenotype. A) Graphical representation of culture protocol and analysis techniques. B) Representative photographs of samples created with TGF‐β1 supplementation and control samples, as well as quantification of the compaction in the x‐direction (*n* = 7 control, *n* = 10 TGFb1, Mann‐Whitney test). C) Maximal intensity projections of second harmonic generation images. D) Quantification of DNA content and HYP content (*n* = 5 control, *n* = 4 TGFb1), Mann‐Whitney test) E). Immunohistochemical staining of αSMA of control and TGF‐β1 samples. F) qPCR data of αSMA, calponin, MYH11, and COL 1. Day 14 samples of donors 3 and 4 are also shown in Figure 4. (*n* = 4 donors with *n* = 3 replicates, Mixed effects model with Bonferroni's multiple comparisons) Scale bars (C) 200 µm, (E) 100 µm, and 25 µm. ^*^ *p* < 0.05; ^**^ *p* < 0.01.; ^***^ *p* < 0.001; ^****^ *p* < 0.0001.

TE‐constructs of the atherosclerotic plaque cap were created with and without the addition of TGF‐β1 supplementation (**Figure** **1**A). Visual inspection of the cultured constructs showed denser tissues in samples supplemented with TGF‐ β1 (Figure 1B). Quantification of the compaction in the x‐direction was also significantly increased in samples supplemented with TGF‐β1 (p < 0.01). Maximum intensity projection images of SHG imaging revealed more collagenous matrix formation in TGF‐β1 supplemented groups compared to control, where a lower amount of collagen could be detected (Figure 1C). The amount of collagenous matrix with and without TGF‐β1 was further quantified using the HYP assay, showing a trend toward increased matrix content (Figure 1D). Additionally, the DNA content was increased in samples with TGF‐β1, indicating an increased cell proliferation, which could have attributed for the increase in matrix (p = 0.02) (Figure 1D). Immunohistochemical staining of αSMA showed positive staining in the samples supplemented with TGF‐β1, while control samples were negative for αSMA (Figure 1E). qPCR analysis of 4 separate MSC donors (Table S1, Supporting Information) showed significant increases in gene expression of αSMA (*p* < 0.0001), Calponin (*p* < 0.001) MYH11 (*p* < 0.01), and COL 1 (*p* < 0.0001) over time, indicating a SMC phenotype after 14 days of culture (Figure 1F). For αSMA the fold change between baseline and day 14 was 8.9 ± 1.5, for Calponin 1.9 ± 0.2 and for COL 1 2.7 ± 0.5. These data indicate the successful deposition of a collagenous matrix as well as differentiation of MSCs toward a SMC‐like phenotype in our TE‐construct by the supplementation of TGF‐β1.

### 2.3 Calcifying Medium Causes Microcalcification Formation in TE‐Cap and Loss of SMC‐Like Phenotype

**Figure 2:**
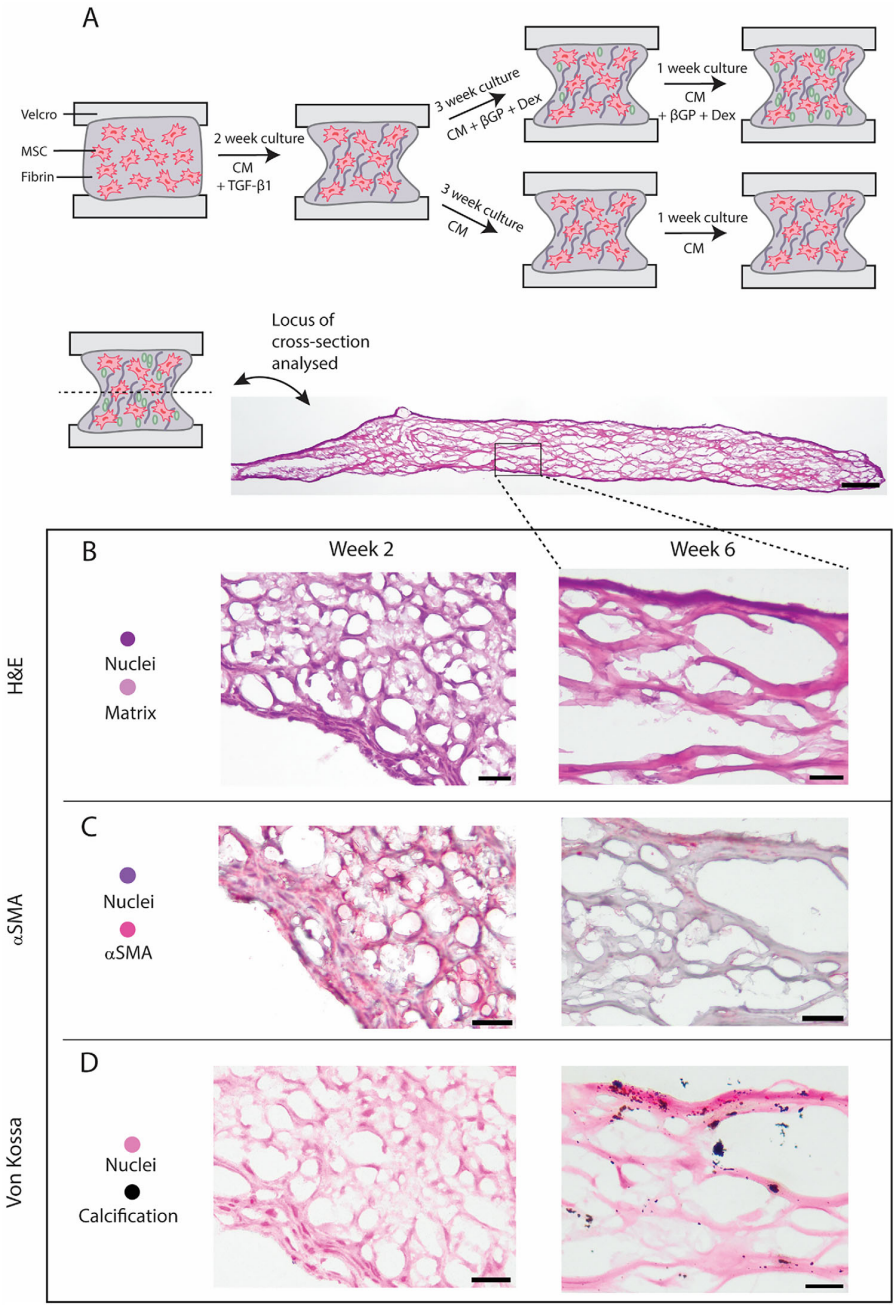
Full protocol for creation of TE‐construct with collagenous matrix and calcification. A) Schematic representation of full culture protocol. B) H&E staining of tissues after 2 weeks and 6 weeks of culture. C) αSMA immunohistochemical staining after 2 and 6 weeks of culture. D) Von Kossa staining after 2 and 6 weeks of culture. Scalebars overview sample: 200 µm (B–D) 50 µm.

After 14 days of supplementation of control medium with TGF‐β1, the medium was switched to initiate calcification formation (**Figure** **2**A) and samples were cultured for an additional 4 weeks. Histological analysis of samples after TGF‐β1 stimulation (week 2) and after culture in calcifying medium (week 6) were compared. H&E staining showed the deposited matrix and cells present at both timepoints (Figure 2B). Immunohistochemical staining of αSMA showed positive staining at week 2, while only limited positive staining at the edges of the sample remained at week 6 (Figure 2D). Von Kossa staining in combination with Nuclear Fast Red again showed cellular presence in the samples, even after 6 weeks of culture. No calcifications were observed at week 2, while microcalcifications were observed throughout the entire depth of the samples at week 6 (Figure 2D; Figure S2A,B,Supporting Information). Calcifications were mainly detected on top of or close to matrix fibers (Figure 2D; Figure S2C, Supporting Information). No microcalcifications were observed in control samples (Figure S2D, Supporting Information). All 3 donors cultured until week 6 (Table S1, Supporting Information) showed matrix formation and microcalcification deposition, with donor differences regarding the amount of matrix and calcification produced (Figure S3, Supporting Information). The matrix structure mimicked the matrix of human fibrous cap tissue (Figure S4, Supporting Information).

**Figure 3:**
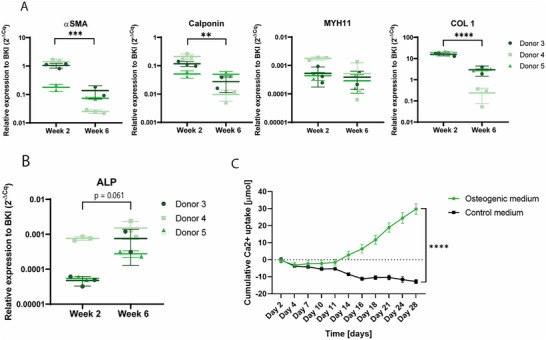
qPCR analysis and calcium assay of TE‐constructs. A) qPCR analysis of genes associated with SMC, αSMA, Calponin, MYH11, and COL 1. (*n* = 3 donors with *n* = 3 replicates for donor 4 and *n* = 2 replicates for donor 3 and 5, Mixed effects model with Bonferroni's multiple comparisons) Day 14 samples of donor 3 and 4 are also shown in Figure 1B) qPCR analysis of ALP. (*n* = 3 donors with *n* = 3 replicates for donor 4 and *n* = 2 replicates for donors 3 and 5, Mixed effects model with Bonferroni's multiple comparisons). C) Calcium assay of the cumulative calcium uptake by the TE‐constructs during culture. Day 2 is the second day of calcifying medium, day 16 of the total culture period. ^*^ *p* < 0.05; ^**^ *p* < 0.01; ^***^ *p* < 0.001; ^****^ *p* < 0.0001.

qPCR analysis of 3 donors (Table S1, Supporting Information) showed significant decreases in gene expression between week 2 and week 6 of αSMA (*p* < 0.001), Calponin (*p* < 0.01) and COL 1 (*p* < 0.0001) (**Figure** **3**A). No significant decrease of MYH11 was shown (Figure 3A), while analysis of ALP showed a trend toward an increase in expression at week 6 (Figure 3B, p = 0.061). Other markers of an osteogenic phenotype, RUNX2 and Msx2, were also analyzed, but showed no significant increase at week 6 (Figure S5, Supporting Information).

After 2 weeks of culture in calcifying medium (total week 4 of culture), the cumulative calcium uptake in the medium was increased when compared to control and this increasing trend was preserved until the end of the culture at week 6 (Figure 3C, *p* < 0.0001). Furthermore, it could be observed that for control samples there was a negative calcium uptake in the medium, which could be due to matrix remodeling during culture, causing calcium ions being released into the medium (Figure 3C).

### 2.4 Microcalcification form at Edges of Samples and Grow During Culture

**Figure 4:**
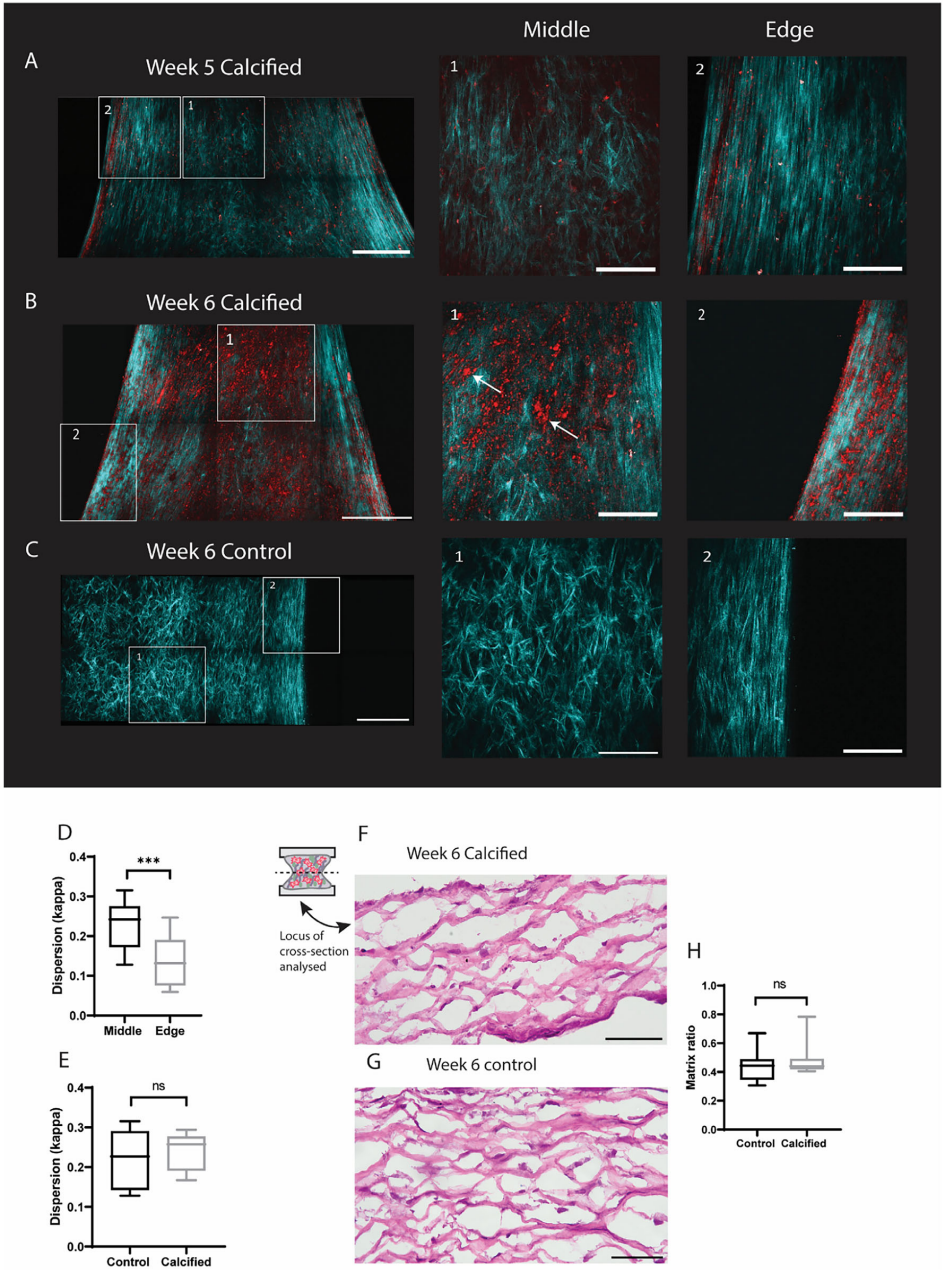
Representative second harmonic generation images of the same sample at timepoint week 5 and week 6. A) SHG maximum intensity projection of a calcified sample with edge and middle regions highlighted. B) SHG maximum intensity projection of the same sample at week 6. C) Control sample at week 6. D) Quantification of anisotropy by dispersion parameter kappa (*n* = 12 z‐stacks for middle and *n* = 11 z‐stacks for edge, unpaired T‐test). E) Quantification of anisotropy by dispersion parameter kappa (*n* = 6 z‐stacks for control and *n* = 6 z‐stacks for calcified, unpaired T‐test). F) H&E staining of calcified sample at week 6. G) H&E staining of control sample at week 6.(H) Quantification of matrix from histological images (*n* = 12 control samples and *n* = 11 calcified samples). Scalebars (A,B): Full image 500 µm, zoomed in images 200 µm, (F,G) 50 µm. ^*^ *p* < 0.05; ^**^ *p* < 0.01; ^***^ *p* < 0.001; ^****^ *p*  0.0001.

Z‐stacks of samples (*n* = 9 calcified at week 5, *n* = 16 calcified at week 6, *n* = 5 control at week 6, Table S1, Supporting Information) were obtained with SHG imaging. 5 samples were imaged both at week 5 and 6, making it possible to compare the collagenous matrix and microcalcification formation within the same sample after an additional week of culture. At week 5, samples showed microcalcification formation especially at the edges of the samples (**Figure** **4**A, region 2). Microcalcifications were also detected throughout the rest of the sample (Figure 4A, region 1). After 1 week of additional culture in calcifying medium, more microcalcifications can be seen throughout the entire tissue (Figure 4B), both in the middle regions (Figure 4B, region 1) and on the edges of the sample (Figure 4B, region 2). Microcalcification size also increased over time, with the biggest particles reaching ≈15 µm in diameter at the end of the culture period (Figure 4B, region 1, arrows). Furthermore, it could be detected that deposited microcalcifications again followed the collagenous matrix as was shown earlier with histology. (Figure S2C, Supporting Information).

When comparing the matrix of calcified samples and control samples with SHG, the same alignment of collagenous matrix could be observed. Both control and calcified samples showed higher anisotropy near the edges of the sample, while the middle region was more isotropic (Figure 4C). The amount of anisotropy in calcified and control samples (*n* = 12 z‐stacks for edge regions and *n* = 11 for middle regions) was further quantified and the measure of dispersion, κ, showed significant higher anisotropy in edge regions compared to control areas (Figure 4D, p = 0.007). No differences were observed in dispersion between control and calcified samples (Figure 4E, p = 0.59). Additionally, histological analysis was performed to further compare the formed matrix between control samples and calcified tissues. H&E staining showed no differences in cellular presence and matrix formation between calcified and control samples (Figure 4F,G). This was further quantified by Orbit analysis of the matrix, showing no significant differences between the matrix ratio of control and calcified samples (Figure 4H).

### 2.5 Microcalcifications Lower TE‐Caps’ Ultimate Tensile Stress and Stiffness

**Figure 5:**
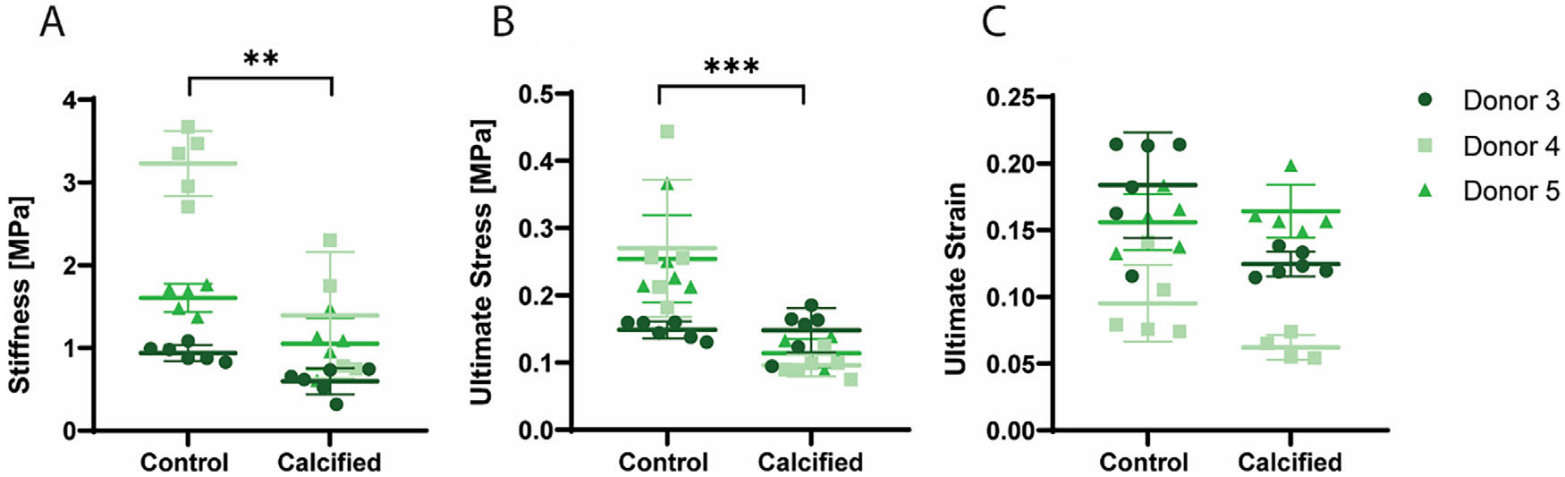
Global mechanics of control versus calcified samples. A) The stiffness at 5% strain, B) The ultimate tensile stress and C) the ultimate strain. (A–C) *n* = 3 donors with *n* = 15 control and *n* = 14 calcified, Mixed effects model with Bonferroni's multiple comparisons. ^*^ *p* < 0.05; ^**^ *p* < 0.01; ^***^ *p* < 0.001; ^****^ *p* < 0.0001.

Mechanical testing until rupture of 3 donors (Table S1, Supporting Information) elucidated global tissue mechanics parameters of control and calcified tissues. Samples that were calcified were significantly less stiff (**Figure** **5**A, *p* < 0.001) compared to control. Furthermore, the ultimate tensile stress (UTS), i.e. the maximum stress measured, was discovered to be significantly lower for the calcified samples compared to the control group (Figure 5B, *p* < 0.0001). No significant difference was observed between experimental groups in the strain corresponding to the UTS, the ultimate strain (Figure 5C).

### 2.6 Increased Calcification Ratio Causes Weakened Mechanical Properties

**Figure 6:**
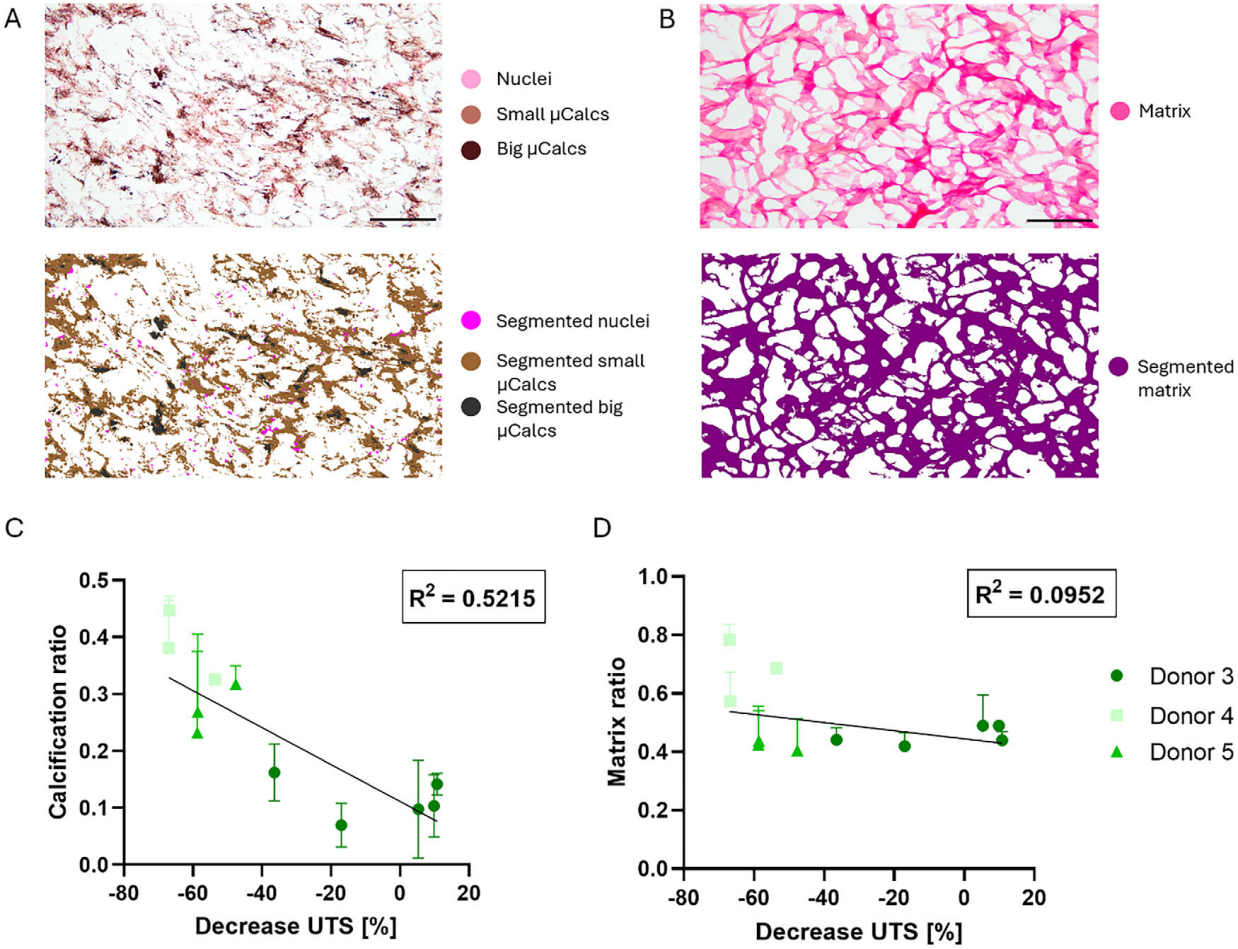
Quantification of histological images and correlation to mechanical parameters. A) Representative von Kossa staining with segmented image, B) Representative Picrosirius Red staining with segmented image, C) Linear regression of the calcification ratio the decrease in UTS, D) Linear regression of the matrix ratio to the decrease in UTS. Scale (A) 200 µm, (B) 100 µm.

Calcification and matrix ratio within the tissue were quantified using the Orbit Analysis Software. Calcification was analyzed from von Kossa‐stained sections in combination with Nuclear Fast Red for cell nuclei. Small microcalcifications, visible as brown deposits, and bigger microcalcifications, seen as black deposits were quantified as well as the ratio of nuclei within the tissue (**Figure** **6**A). Quantification was verified by comparing the segmented image to the original image (Figure 6A). The total calcification ratio was obtained by combining data from the small and big microcalcifications. For Picrosirius Red stained sections, the matrix was the only component to be quantified and segmented within the images (Figure 6B).

The relationship between the quantified histological images and mechanical properties, stiffness and UTS, was analyzed by linear regression. The analysis of the decrease in UTS to calcification ratio showed a correlation with an R^2^ of 0.5215 (Figure 6C, p < 0.001), while for the matrix this was only 0.0952 (Figure 6D, p = 0.08). Additionally, the correlation between the collagen and calcification ratio within the same samples was analyzed by a Spearman correlation and no significant correlation was found between those (Spearman = 0.42, p = 0.20).

### 2.7 The Effect of Microcalcifications on TE‐Caps’ Cyclic Loading Behavior

**Figure 7:**
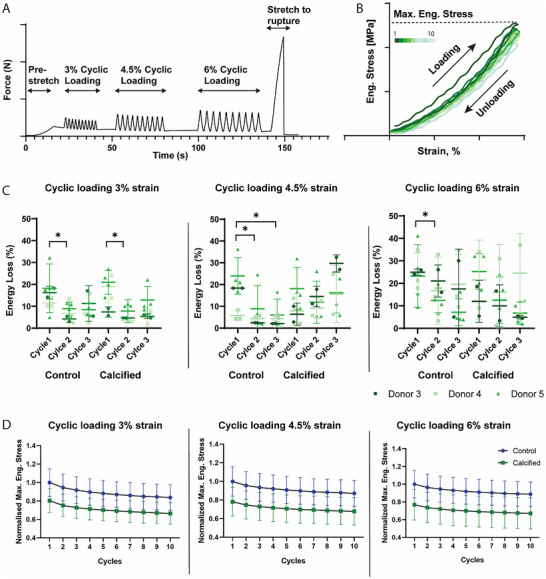
Cyclic loading at 3%, 4.5%, and 6% strain for calcified and control samples. A) Representative image of loading protocol with pre‐stretch, cyclic loading, and loading until rupture. B) Graphical image of obtained hysteresis C) Energy loss quantification of the first 3 cycles per cyclic loading condition, *n* = 10 samples control and *n* = 10 samples calcified, Mixed effects model with Bonferroni's multiple comparisons D) The normalised maximum engineering stress that is reached at each cycle. ^*^ *p* < 0.05; ^**^ *p * < 0.01; ^***^ *p* < 0.001; ^****^ *p* < 0.0001.

Cyclic loading was performed for 10 cycles at consecutive 3%, 4.5%, and 6% strain (**Figure** **7**A). Hysteresis loops were created, where the loading and unloading phase could be visualized and the maximum engineering stress during each cycle could be obtained (Figure 7B). The TE‐constructs displayed time and load dependent properties. The hysteresis of the first loop was observed to be different from the rest of the loops, which is why the energy loss of the first three cycles was calculated. When looking at the 3% strain, it can be seen that the energy loss decreases significantly between cycle 1 and 2 for both control samples (p = 0.02) and calcified samples (p = 0.03)(Figure 7C). For the 4.5% strain a similar pattern could be observed for the control samples (p = 0.02 for cycle 1 to 2 and p = 0.02 for cycle 1 to 3) while the calcified samples showed no significant decrease (Figure 7C). Lastly, this pattern was also observed during the third phase of cyclic loading, with 6% strain, for the control samples (p = 0.04 for cycles 1 to 2), with no significant differences being observed for the calcified group. No significant differences were observed between cycles 2 and 3 for both conditions, suggesting the biggest decrease in energy dissipation is reached during the first 2 cycles of loading (Figure 7C).

Similar to the decrease in energy dissipation, the maximum stress per cycle that was reached per cycle decreases, with the biggest decrease happening in the first three cycles, after which the stress stabilizes (Figure 7D). Furthermore, it can be observed that the maximum engineering strain of the calcified samples is lower than control for all donors at all straining conditions (Figure 7D).

## 3 Discussion

Within this study, we have created an in vitro TE model of the human atherosclerotic plaque cap, with biologically deposited collagenous matrix and microcalcifications. This model can shed light on the complex interplay between collagen, microcalcification formation, cellular phenotype and mechanical behavior in plaque vulnerability. MSCs were differentiated with TGF‐β1 into a smooth muscle cell (SMC)‐like phenotype. During this differentiation stage, a collagenous matrix was deposited and afterward SMC‐like cells underwent mineralization and formed microcalcifications within TE plaque caps. These microcalcifications followed the collagen matrix, mainly on the edges of the samples, and increased in amount and size over time. Mechanical characterization using tensile testing until rupture showed the effect of microcalcifications on global tissue mechanics, as the ultimate stress at rupture and stiffness of the tissue was lowered significantly.

During the first 2 weeks of culture, MSCs were differentiated into SMC‐like cells with TGF‐β1 supplementation. This differentiation has previously been performed in monolayer experiments with human umbilical cord‐derived MSCs,^[^ ^37^ ^]^ human adipose tissue‐derived MSCs^[^ ^40^ ^]^ and human bone marrow‐derived MSCs.^[^ ^38^ ^]^ Within our study, differentiation from paediatric bone marrow‐derived MSCs was attempted in a 3D‐culture system. Fold changes were observed to be in the same range as in previously stated literature, indicating successful differentiation toward a SMC‐like phenotype. Additionally, TGF‐β1 caused increased proliferation and matrix deposition in our samples. TGF‐β1 has been shown to have both negative and positive effects on cellular proliferation of MSCs, dependent on the dosage, differentiation stage and timing.^[^ ^44^ ^]^ TGF‐β1 can induce SMAD3‐dependent proliferation and matrix secretion in both MSCs and SMCs.^[^ ^44^, ^45^ ^]^ Furthermore, differentiated SMCs are able to reversibly modulate their phenotype and change between differentiation, proliferation and matrix synthesis.^[^ ^46^ ^]^ After 2 weeks of culture to stimulate SMC‐differentiation and collagen deposition, medium was changed toward a calcifying medium to induce microcalcification formation within the TE‐constructs. With regard to qPCR data, it was seen that cells lost most of their SMC lineage markers, which has been observed in a previous study where aortic SMCs underwent mineralization in vitro.^[^ ^47^ ^]^ This could indicate that cells differentiated again from an SMC‐like phenotype toward a more mineralizing phenotype, as intended. Gene expression of ALPL showed an upregulated trend, although not significantly, while no differences were observed in Runx2 and Msx2 expression. Both of these transcription factors are involved in calcification processes in smooth muscle cells in atherosclerotic fibrous caps.^[^ ^20^, ^48^, ^49^, ^50^ ^]^ However, it is known that these are transcription factors that might peak in the early differentiation toward an osteogenic phenotype.^[^ ^51^ ^]^ The expression is lowered in the later stages of differentiation, which might be the reason why no differences were observed in this study. Future studies could focus on analyzing multiple timepoints of osteogenic differentiation and calcification. Since MSCs were used in this study, it could be argued that cells which potentially did not fully differentiate toward a SMC phenotype, differentiated directly from MSC toward an osteoblast in the calcifying environment^[^ ^52^ ^]^ and thus did not transdifferentiate from SMCs. In current literature, it is hypothesized that bone formation, via osteoblasts, and ectopic vascular calcification resemble one another. However, it has been shown that osteoblasts and SMCs can still show significant differences in their calcification behavior.^[^ ^53^ ^]^ Where osteoblasts often form large mineralized bone nodules, calcifying SMCs form small discrete regions of calcification.^[^ ^53^ ^]^ Future studies on this model could focus on elucidating the exact cellular phenotypes present at different stages of the culture at a single cell resolution to further explain the calcifying mechanism.

Second harmonic generation microscopy facilitated imaging of the collagenous matrix as well as the microcalcification formation during culture. Microcalcifications predominantly formed along the edges of the sample. Multiple hypotheses to why this is the case can be proposed. Firstly, from SHG imaging it could be observed that collagen alignment was highest in the edges of the samples. It has been shown that microcalcification use the collagenous matrix as a scaffold,^[^ ^54^ ^]^ which was also observed in our study, highlighting the role between collagen alignment and calcification formation. Various other observational studies have shown that fiber alignment may enhance tissue calcification with calcification progressing along the aligned fibers, such as in immature chick tibia^[^ ^55^ ^]^ and in calcific aortic valve disease.^[^ ^56^ ^]^ Since these differences in calcification initiation location were seen within the samples, this highlights the importance of the hypothesis that calcification can be driven by composition of the fibrous cap. The mechanisms by which collagen alignment induces mineralization could possibly be attributed to cellular migration toward anisotropic regions and consequent formation of microcalcifications.^[^ ^57^ ^]^ A second hypothesis, related to collagen fiber alignment, is the fact that there can be differences in tension within the tissue during the culture period, due to compaction of the tissue altering the loading at the edges of the sample. Differences in mechanical loading and the effect on calcification can possibly be linked to Wolff's Law, which states that bone growth is affected by mechanical stimulation to optimize their structure.^[^ ^58^ ^]^ In porcine aortic valves it has been shown that elevated stretch elicited a stronger calcification response.^[^ ^59^ ^]^ Furthermore, in tissue‐engineered tendon, alkaline phosphatase activity was increased in highly strained groups.^[^ ^60^ ^]^ Higher amounts of stretch could induce SMCs to transdifferentiate toward an osteoblast‐like phenotype and encourage calcification.^[^ ^61^ ^]^ In addition, mechanical stretch is known to induce apoptosis in SMCs^[^ ^62^ ^]^ and regions with increased apoptosis have been shown to correlate with elevated calcification response,^[^ ^59^ ^]^ as calcification might use apoptotic cells and cellular debris as an anchoring point. Thus, tension during culture and collagen alignment could play a role in the onset of microcalcification formation and elucidating the specific role of these in vascular calcification could provide additional biomarkers for plaque vulnerability. Future studies could thus focus on assessing the effect of cap composition on calcification in an osteogenic and non‐osteogenic environment. Highly anisotropic and isotropic tissues could be created by adapting the location of the Velcro strips within the model. These models could then be cultured with varying concentrations of osteogenic supplements to assess the dominant factor in calcification formation.

During mechanical testing, the TE‐constructs were strained until rupture to obtain global mechanical properties of the tissue. We detected that samples with microcalcifications had decreased UTS, which could be explained by the fact that microcalcifications can be seen as local stress accumulators in atherosclerotic plaque caps, as has been shown in various computational models.^[^ ^11^, ^63^, ^64^ ^]^ In previous work of our group, hydroxyapatite microparticles within a collagenous matrix also showed a decrease in UTS,^[^ ^65^ ^]^ possibly due to influencing the dispersion of collagen fibers, as it was shown that local collagen isotropy was increased in regions of hydroxyapatite particles.^[^ ^65^ ^]^ In the current study, formed microcalcifications did not alter collagen alignment. This could be due to the particles being smaller than 15 µm, while only bigger particles up to 50 µm, which were present in the study with hydroxyapatite particles, cause these changes in isotropy. The decrease in UTS might however still be due to the interplay with collagen fibers, as hydroxyapatite crystal growth might create fissures in the matrix.^[^ ^66^ ^]^ In a study on the calcification on tendon fascicle bundles it was shown that calcific deposits change the tendon structure, by forming within the tendon fibers, modifying the collagen matrix and leading to damage and impairment of the mechanical function.^[^ ^67^ ^]^ In our samples, calcification might also cause local defects within the collagenous matrix, consequently creating regions more prone to rupture and lowering the UTS and stiffness of the tissue, thus increasing the rupture risk of TE constructs and atherosclerotic plaques. In order to validate these findings, clinical *ex vivo* samples with similar cap components to the TE samples could be imaged and mechanically tested. To be able to further translate the results of this study to the clinic, imaging modalities capable of detecting these small calcifications in vivo are needed. ^18^F‐sodium fluoride positron emission tomography is able to non‐invasively detect high risk regions of microcalcifications which can be linked to unstable plaques.^[^ ^68^ ^]^ Additionally, Optical Coherence Tomography (OCT) provides a high‐resolution, invasive imaging modality able to assess both microcalcifications and macrophages.^[^ ^69^ ^]^ The detection and characterization of microcalcifications could improve early detection and risk‐assessment to prevent acute cardiovascular events.

Additionally, differences regarding the mechanical properties of the tissues were linked to the matrix production and calcifying potential of the cells. This correlation was based on the fact that donor differences caused differences in the amount of calcification and matrix production. The different donors could be visualized in subgroups within the correlation, with a donor which formed more calcification also decreasing stronger in UTS. It was shown that an increase in calcification ratio within the tissue was correlated to a decrease in UTS, while this relationship was not significant for the amount of matrix in the tissue, suggesting that the amount of microcalcifications could be a key player in plaque rupture, which was not the case for matrix content. With regard to the amount of calcification, it has been shown before in computational models that an increase in particle clustering can enhance the local tissue stress and thus the rupture potential of atherosclerotic plaques.^[^ ^11^, ^17^ ^]^ Furthermore, in an experimental study by Cardoso, higher concentrations of micro‐beads in a silicone‐based model showed a decrease in UTS,^[^ ^70^ ^]^ further supporting our data that an increase in calcified particles can increasingly weaken the mechanical properties of the constructs. However, the calcification content could only partially explain the mechanical properties of the tissue. Other characteristics of the calcifications within the TE constructs could additionally play a role in mechanics, such as the spacing between particles,^[^ ^71^ ^]^ location^[^ ^72^ ^]^ and shape.^[^ ^11^, ^64^ ^]^ While previously collagen content was thought to be one of the main determinants of plaque rupture, recent studies have shown that there is a poor correlation between collagen content and the ultimate stress.^[^ ^73^ ^]^ Other collagen characteristics, such as fiber orientation,^[^ ^73^ ^]^ crosslinking^[^ ^74^ ^]^ and collagen type^[^ ^75^ ^]^ could further determine the mechanical behavior of plaques.^[^ ^76^ ^]^ Future studies with this model could focus on assessing the correlation between microcalcification amount and tissue mechanics more in depth, by specifically altering the amount of microcalcifications formed.

Hysteresis loops obtained during cyclic loading of our TE‐constructs with and without calcifications provided data on the mechanical characteristics of our model in relation to atherosclerotic plaque tissue. It is known that soft biological tissues, such as vessels, can be characterized by stress hysteresis and softening effect under cyclic loading.^[^ ^77^ ^]^ This softening effect, which is expressed as a decrease in the stress under load and has previously been seen in carotid atherosclerotic plaque samples,^[^ ^78^ ^]^ can also be seen in our tissues, where the maximum engineering stress decreased after the first cycle of cyclic loading. This material softening could be due to microstructural changes within the collagenous matrix of the tissue or damage accumulation, leading to a reduction in ability to withstand stress.^[^ ^79^ ^]^ As the maximum stress that was reached was lower for the calcified samples, this could indicate that the microcalcifications caused more local microstructural changes, such as microcracks or weakened bonds, to the collagenous matrix, causing a lower ability to withstand the stress. Additionally, the softening effect could also be explained by poroviscoelastic effect induced by a changing ratio between the solid and the fluid phase. However, this ratio was not quantified, so no conclusive statements can be made about this phenomenon. Biological tissues undergoing tissue softening are also characterized by a decrease in hysteresis area.^[^ ^77^, ^80^, ^81^ ^]^ This effect was also seen in our tissues, especially in the first two cycles, possibly due to rearrangement of the collagen fibers, after which no further changes to the matrix are being made during loading, causing the hysteresis area to stabilize during the rest of the cyclic loading protocol. Additionally, the decrease in energy loss was significant for all cyclic loading protocols in control samples, while this was not the case for calcified samples. This reduced softening effect in calcified samples could be due to the microdamage within these samples due to the calcifications possibly interfering with the collagen network. Further analysis of the mechanical behavior of calcified and non‐calcified samples under cyclic loading could contribute to the understanding of the materials properties of the atherosclerotic plaque cap in the dynamic environment of the cardiovascular system and serve as a biomarker for plaque rupture risk assessment.

A limitation of this study is the relative simplicity of the created samples when compared to the complexity of an atherosclerotic plaque lesion. These atherosclerotic plaques consist of a lipid‐rich necrotic core, inflammatory cells such as macrophages and multiple other cell‐types and phenotypes within the cap.^[^ ^3^ ^]^ The current model only includes one cell‐type, which deposits the collagenous matrix and microcalcifications. This simplicity makes it possible to assess the effect of microcalcifications within a collagenous matrix on the mechanical properties, while limiting the possibility of bias due to other components. Future studies could focus on further elaborating this model by creating a co‐culture of multiple cell types, including multiple SMC phenotypes, adding a lipid‐rich necrotic core or macrophages and other inflammatory cells to mimic the atherosclerotic plaque even further.^[^ ^76^ ^]^ However, it should be kept in mind that through drastically increasing the complexity, the model will become more variable, making it more difficult to interpret results without over‐ or underemphasis of the components involved.

The established model could be used to further elucidate the interplay between important components of the atherosclerotic plaque with regard to tissue mechanics. Furthermore, cellular interactions with their microenvironment, such as the collagenous matrix and deposited microcalcifications can be studied on deeper gene expression level, as well as proteomics, mechanobiology and matrix remodeling. Additionally, cellular mechanisms involved in the differentiation from MSC to SMC and calcifying SMC can be assessed, such as the presence and involvement of calcifying extracellular vesicles.^[^ ^82^ ^]^ The model could possibly serve as a platform for drug screening to identify potential therapeutic targets, such as anti‐calcification agents, for stabilizing vulnerable plaques.

## 4 Conclusion

In conclusion, the current study offers valuable insights into the role of microcalcifications in the atherosclerotic plaque cap and their impact on biomechanical stability. A tissue‐engineered model was created by differentiating MSCs to a SMC‐like phenotype and inducing calcification afterward. By live imaging during culture, the progression of microcalcification formation could be characterized, and mechanical testing showed that the presence of microcalcifications led to alterations in the mechanical properties, as evidenced by reduced ultimate stress and stiffness. These findings underscore the importance of considering microcalcifications as critical contributors to plaque vulnerability and suggest a potential target for therapeutic interventions aimed at mitigating cardiovascular events associated with plaque rupture.

## 5 Experimental Section

### Preculture of MSCs

Human bone marrow MSCs (*n* = 5 donors in total, see Table S1 (Supporting Information) for information on which donors were used for which analysis) were isolated from leftover/waste iliac crest bone chips obtained from paediatric patients undergoing alveolar bone graft surgery (*n* = 4 male patients, age between 10 and 12). All samples were harvested with consent for the use of surgical waste material with a possibility for parental opt‐out and approved by the Medical Ethics Review Committee at the Erasmus MC Medical Ethical Committee(MEC‐2014‐106). Cells were expanded in αMEM (Gibco, Thermo Fisher Scientific, Breda, the Netherlands) containing 10% heat inactivated foetal bovine serum (FBS, Sigma‐Aldrich, St. Louis, USA), supplemented with 50 µg mL^−1^ gentamycin, 1.5 µg mL^−1^ fungizone, 25 µg mL^−1^ L‐ascorbic acid 2‐ phosphate (Sigma–Aldrich, St. Louis, USA) and 1 ng mL^−1^ fibroblast growth factor‐2 (Instruchemie B.V., Delfzijl, The Netherlands) in a humidified environment at 37 °C and 5% CO_2_. They were expanded until 80% confluency after which they were passaged to passages 3 to 5 for experimental set‐ups.

### Monolayer Cell Culture—MSC to SMC Differentiation

MSCs were seeded at 2300 cells cm^−2^ in 6‐wells plates and cultured in a variety of culture media, based on previous literature,^[^ ^36^, ^37^, ^40^, ^83^ ^]^ to assess the optimal culture medium for SMC differentiation. Cells were cultured either in αMEM (Gibco, Thermo Fisher Scientific, Breda, the Netherlands), high glucose (HG) DMEM (Gibco, Thermo Fisher Scientific, Breda, the Netherlands) or DMEM (Gibco, Thermo Fisher Scientific, Breda, the Netherlands) with either 1% or 10% FBS (Sigma‐Aldrich, St. Louis, USA), all supplemented with 50 µg mL^−1^ gentamycin, 1.5 µg mL^−1^ fungizone, 25 µg mL^−1^ L‐ascorbic acid 2‐ phosphate (Sigma‐Aldrich, St. Louis, USA) and 5 ng/ml TGF‐β1. Cells were cultured for 6 days after which they were harvested for PCR analysis in a humidified environment at 37 °C and 5% CO2.

### Monolayer Cell Culture—SMC Calcification

After the 6 day stimulation with TGF‐β1, medium was changed toward a mineralizing medium, consisting of HG DMEM (Gibco, Thermo Fisher Scientific, Breda, the Netherlands) containing 10% heat inactivated FBS (Sigma‐Aldrich, St. Louis, USA), supplemented with 50 µg mL^−1^ gentamycin, 1.5 µg mL^−1^ fungizone, 25 µg mL^−1^ L‐ascorbic acid 2‐ phosphate (Sigma–Aldrich, St. Louis, USA), 100nm dexamethasone (Sigma–Aldrich, St. Louis, USA) and 10 mm β‐Glycerophosphate (BGP, Sigma‐Aldrich, St. Louis, USA). Samples were harvested after 15 additional days of culture (total culture period 24 days) and used for histological analysis.

### Tissue‐Engineered Plaque Caps—Creation of TE Plaque Caps with MSCs

TE plaques were created following the methodology described previously.^[^ ^84^ ^]^ To summarize, MSCs (1.5×10^6^ cells mL^−1^) were seeded in 1.5×1.5 cm‐sized fibrin gels, a suspension of bovine fibrinogen (10 mg mL^−1^, Sigma F8630) and bovine thrombin (10 U mL^−1^, Sigma T4648), cast between two Velcro strips (1.5 cm long). Samples were left to solidify at 37 °C for 30 min, after which medium was added. After 1 h of culture, part of the samples (*n* = 12) were harvested for qPCR analysis at baseline.

### Tissue‐Engineered Plaque Caps—MSC to SMC Differentiation and Calcification

After seeding, the samples were cultured in a SMC differentiation medium (chosen after monolayer experiments) for 14 days consisting of αMEM (Gibco, Thermo Fisher Scientific, Breda, the Netherlands) containing 10% heat inactivated FBS (Sigma‐Aldrich, St. Louis, USA), supplemented with 50 µg mL^−1^ gentamycin, 1.5 µg mL^−1^ fungizone, 50 µg mL^−1^ L‐ascorbic acid 2‐ phosphate (Sigma–Aldrich, St. Louis, USA) and 5 ng mL^−1^ TGF‐β1. For the first 7 days of culture, ε‐Amino Caproic Acid (ε‐ACA, 1 mg mL^−1^, Sigma) was added to prevent fibrin break‐down.^[^ ^85^ ^]^ After this 14‐day differentiation, a part of the samples (*n* = 12) were harvested for qPCR analysis, while the other samples (*n* = 28 calcified and *n* = 18 control) were kept in culture for an additional 28 days. The medium was changed from SMC differentiation to the mineralising medium (same as in monolayer experiments). Control samples were cultured in the same medium, without the supplementation of dexamethasone and β‐Glycerophosphate.

### Analysis—DNA Content

Samples (*n* = 4 control and *n* = 5 with TGF‐β1) used for DNA determination were lyophilized, weighed to determine the dry weight of the samples, and digested in a papain digestion buffer (100 mm phosphate buffer (pH 6.5), 5 mm L‐cysteine (C‐1276), 5 mm ethylene‐di‐amine‐tetra‐acetic acid (EDTA, ED2SS), and 140 *µg* mL^−1^ papain (P4762), all from Sigma‐Aldrich. Each sample was mixed with 300 *µ*L of digestion buffer in a new Eppendorf tube and placed at 60 °C for 16 h for digestion. Digested samples were vortexed and centrifuged (12 000 rpm, 10 min), and subsequently the DNA content was quantified using the CyQUANT kit (Invitrogen C7026, Thermo Fisher, Waltham, USA), following the manufacturer's instructions. DNA content is represented relative to dry weight of the samples.

### Analysis—Hydroxyproline Assay

To quantify collagen content, the hydroxyproline (HYP) assay was performed (*n* = 4 control and *n* = 5 with TGF‐β1). The digested samples were hydrolyzed using 16m sodium hydroxide. Subsequently, HYP content was quantified using the Chloramin‐T assay, including trans‐4‐hydroxyproline as a reference (Sigma, H5534).^[^ ^86^ ^]^ The HYP content was then normalized for the dry weight of the samples.

### Analysis—Calcium Uptake Assay

Mineralization was monitored during culture by determining the calcium uptake from the culture medium. At each medium change (3 times per week) 200 µL of culture supernatant was collected from the TE samples and stored at −20°C. The calcium concentration was calculated using a standard curve of 0–3.0mM CaCl_2_ (Sigma–Aldrich, St. Louis, USA) in calcium‐free DMEM (Thermo Fisher, Waltham, USA). 10 µL of sample was mixed with 100 µL of a calcium reagent (1 + 1 mix of 1 m ethanolamine pH 10.5 and 0.35 mm o‐cresolphthalein complexone, 19.8 mM 8‐hydroxyquinoline and 0.6 M hydrochloric acid, all from Sigma‐Aldrich, St. Louis, Missouri, USA). The optical density was measured with the VersaMax spectrophotometer (Molecular Devices, San Jose, California, USA) at a wavelength of 570 nm. The cumulative calcium uptake by the TE‐constructs was calculated by subtracting baseline values, wells with empty medium, and correcting for the amount of medium added per well.

### Analysis—Gene Expression Analysis

The monoculture MSCs were harvested on day 6 (after SMC differentiation), lysed by the addition of 500 µl of RLT lysis buffer (Qiagen) with 1:10 β‐mercaptoethanol and stored at −80°C. For TE plaque caps the sample was first disrupted using a tissue dismembrator (mikro‐dismembrator S, B. Braun BioTech International, Melsungen, Germany). In short, tissues were frozen in liquid nitrogen and added to a frozen chamber with a metal ball. The chamber was secured in the tissue dismembrator and disrupted at 2800 rpm for 1 min. Afterward RNA STAT‐60 (Gentaur) was added, the samples defrosted and the lysate mixed with chloroform in a 1:5 ratio to the lysate. After centrifuging at 8,000g a 3‐phase solution was created and the aqueous phase was transferred to a new tube. For RNA isolation of both the monolayer as the TE samples, the lysed cell mixture was mixed with an equal volume of 70% v/v ethanol and loaded into the Epoch Life Science Mini Spin columns (#1940‐250, Epoch Life Science). Isolation was performed according to the manufacturers’ protocol, and RNA was quantified using a fluorometer (DSS‐100 Series, DeNovix, Wilmington, USA). Afterward, cDNA isolation was performed according to instructions of the manufacturer of the RevertAid First Strand cDNA Kit (Thermo Fisher Scientific, Waltham, Massachusetts, USA). Gene expression was performed by qPCR analysis using either the qPCR Mastermix (TaqMan Universal PCR Mastermix (Thermo Fisher Scientific, Waltham, Massachusetts, USA)), or the qPCR Mastermix Plus for SYBR Green (EUrogentec, Seraing, Belgium). Signal was measured using the Bio‐Rad CFX96 Real‐Time PCR Detection system (Bio‐Rad, Hercules, California, USA). The primers used for analysis were αSMA (forward: 5’‐CGTGTTGCCCCTGAAGAGCAT‐3’, reverse: 5’‐ACCGCCTGGATAGCCACATACA‐3’), Calponin (forward: 5’‐TTGAGGCCAACGACCTGTTT‐3’, reverse: 5’‐TTTCCGCTCCTGCTTCTCTG‐3’), Myosin Heavy Chain 11 (MYH11) (forward: 5’GTCCAGGAGATGAGGCAGAAAC‐3’, reverse: 5’‐GTCTGCGTTCTCTTTCTCCAGC‐3’), COL 1 (forward: 5’‐CAGCCGCTTCACCTACAGC‐3’, reverse: 5’‐TTTTGTATTCAATCACTGTCTTGCC‐3’), ALPL (forward: 5’‐GACCCTTGACCCCCACAAT‐3’, reverse: 5’‐GCTCGTACTGCATGTCCCCT‐3’), RUNX2 (forward: 5’‐ACGTCCCCGTCCATCCA‐3’, reverse: 5’‐TGGCAGTGTCATCATCTGAAATG‐3’), and Msx2 (forward: 5’‐CGGAAAATTCAGAAGATGGAGCG‐3’, reverse: 5’‐ CGGCTTCCGATTGGTCTTGTGT‐3’). The best housekeeper Index (BKI) was calculated from expression of two genes, being Ubiquitin C (UBC) (forward: 5’‐ ATTTGGGTCGCGGTTCTTG‐3’, reverse: 5’‐ TGCCTTGACATTCTCGGATGGT‐3’) and Beta‐2‐Microglobulin (B2M) (forward: 5’‐ TGCTCGCGCTACTCTCTCTTT‐3’, reverse: 5’‐ TCTGCTGGATGACGTGAGTAAAC‐3’). Subsequently, gene expression was calculated relative to the BKI, making use of the ΔΔCt method where Gene Expression = 2−ΔCq and ΔCq = CqSample—CqBKI.

### Analysis—Imaging of TE Plaque Caps

After the culture period, the TE samples (*n* = 5 at week 2, *n* = 9 calcified at week 5, *n* = 16 calcified at week 6, *n* = 5 control at week 6) were rinsed with phosphate buffered saline (PBS, Gibco, Thermo Fisher Scientific, Waltham, USA). The samples which were calcified were incubated with a hydroxyapatite‐targeting probe (IVISense Osteo 680 Fluorescent Probe, Osteosense, PerkinElmer), diluted 1:200 in PBS at 4°C for 48 h. Additionally, 2 control samples were incubated with this probe, to verify no calcifications formed in control samples. Following incubation, samples were rinsed with PBS, and were pinned to a silicone‐filled (Sylgard 184, VWR, Germany) petri‐dish with sterile surgical needles (Sterican, B. Braun Medical BV, Oss, The Netherlands). PBS was added to fully submerge the sample. A multiphoton microscope (TCS SP5 Confocal, Leica, Germany) with a Chameleon Ultra multiphoton laser (710–1040nm) (Coherent, USA) was used to visualize collagen architecture and microcalcifications. First, a bright‐field tile scan of the sample was made. Next, second harmonic generation (SHG) using two‐photon microscopy (excitation of 880nm) was employed to image collagen fibers in combination with confocal microscopy of the calcified particles (excitation of 680 nm). Z‐stacks (tile size 739×739µm, step size 3 µm, pixel size 1.4 µm x 1.4 µm) to a depth of ≈200 µm were collected in various areas of the TE‐constructs. For further data analysis, the maximum intensity projection (MIP) was obtained and analyzed using the Fiblab software^[^ ^87^ ^]^ to extract the dispersion (κ) of the fibers, as a measure of the (an)isotropy in various regions of the engineered tissues.

### Mechanical Characterization: Uniaxial Tensile Testing

Uniaxial tensile tests were performed after SHG imaging to assess the effect of calcification on TE caps’ mechanical properties (*n* = 16 control samples and *n* = 17 calcified samples). Before testing, samples were rinsed in PBS and imaged with a high frequency, high spatial resolution ultrasound system (VEVO 3100, FUJIFILM VisualSonics, Canada) using a linear transducer (MX550) to assess the dimensions of the sample. For uniaxial tensile testing, a custom‐designed set‐up^[^ ^88^, ^89^ ^]^ equipped with a 20N load cell (LCMFD‐20N, Omega Engineering, USA) was used. The samples were placed in the uniaxial tensile tester using the Velcro constraints, and the tests were performed while the samples were submerged in PBS at 37 °C. A pre‐load of 0.05 N was applied to remove tissue slack after which the cyclic loading protocol was started. Cyclic loading was performed at 3%, 4.5%, and 6% strain with 10 loading cycles per strain condition. Strain values for cyclic loading were selected after pilot experiments to assess average maximum strain in the tissues. Afterward, the final uniaxial tensile stretching cycle until complete rupture was performed at a strain rate of 200%/min.

From the cyclic loading data hysteresis loops were obtained. The maximum stress at each cycle was calculated as well as the energy dissipation from the area between the loading and unloading curves as a percentage of the loading energy. The engineering stress‐strain behavior of the samples for the final uniaxial tensile stretch cycle was calculated, and ultimate tensile stress and strain, and the tangential modulus at 5% strain, were assessed.^[^ ^90^, ^91^ ^]^ Cross‐sectional area measurements from the ultrasound scans were used for stress calculations and gauge length for the strain measurements. For the ultimate tensile stress, the calcified samples were normalized to control samples within the same donor to obtain the percentual change.

### Histological Analysis

6 µm thick cryosections, cut in the z‐direction (i.e. the depth of the samples), were analyzed to evaluate global collagen matrix and the formation of microcalcifications throughout the samples. Global matrix features could be assessed using a hematoxylin & Eosin (H&E) staining, while microcalcifications were stained with von Kossa and the collagenous matrix was analyzed with Picrosirius Red. For the H&E staining, samples were first stained in hematoxylin for 5 min. Afterward they were washed in tap water and stained in eosin for 45 s and dehydrated. For the von Kossa staining, the sections were stained in a silver nitrate solution for 30 min, rinsed in Milli‐Q, counterstained with Nuclear Fast Red and dehydrated. For the Picrosirius Red staining, the samples were staining in Picrosirius Red solution for 60 min, after which they were washed in acetic acid and dehydrated in a series of ethanol. Stained sections were embedded in enthalan and imaged with brightfield microscopy (Olympus BX50).

Orbit Image Analysis software was used to classify the von Kossa staining for the ratio of calcification to the voids within the tissue and for the ratio of matrix to voids in Picrosirius Red stained sections.^[^ ^92^ ^]^ For each classification model, two images per donor with six regions per image (*n* = 36 regions in total) were used to define the training set with the goal of covering the entire variability of the tissue structures and donors. Regions were manually drawn to select regions of either calcification particles, matrix, cell nuclei or voids. The trained model was then used to classify these components in the total data set (*n* = 3 donors, *n* = 3 samples per donor). Data was further analyzed and correlated to the stiffness and ultimate stress values obtained from mechanical tensile testing.

### Immunohistochemistry

6 µm thick cryosections were fixed in acetone and blocked and permeabilized using 3% Normal Goat Serum (NGS, Southern Biotech, Birmingham, USA), 1% Bovine Serum Albumin (BSA, Thermo Fisher, Waltham, USA), 0.5% Triton‐X‐100, 0.5% Tween in PBS for 2h at room temperature. Pre‐incubation was done with 10% NGS in PBS/1% BSA for 30 min. Sections were incubated with anti‐Smooth Muscle Actin α (αSMA, Sigma, #A2547, 0.37µg mL^−1^ in PBS/1% BSA) for 1h at room temperature. The sections were then incubated with 1:50 biotinylated goat‐anti‐mouse antibody (Biogenex, Fremont, USA) for 30 min, followed by incubation with 1:50 streptavidin‐AP (Biogenex, Fremont, USA). Staining was revealed by incubation with Neu Fuchsin substrate (Chroma, 1g/25mL 2m HCl) and sections were mounted with mounting solution (VectaMount, Vector Laboratories, Newark, USA). Afterward the sections were visualized with brightfield microscopy (Olympus BX50).

### Statistical Analysis

Statistical analyses were performed using Prism (GraphPad, La Jolla, CA, USA). A Shapiro‐Wilk test was performed for normality. For the pre‐processing of the data, samples were tested for normality. In case of proven normality and the comparison of two experimental groups with only 1 donor, an unpaired T‐test was performed, and a nonparametric Mann‐Whitney U test otherwise. For all multiple comparisons, a mixed effects model with Bonferroni correction was used. The different conditions (control vs. calcified samples) were considered as a fixed parameter and the donor as a random factor. By incorporating the donor as a random effect, potential confounding influences of between‐donor variability was accounted for. Replicates in this study refer to individual tissues and were treated as independent observations. Only one sample per tissue‐engineered construct was assessed. For the analysis of quantified histological data in relation to the mechanical parameters, a linear regression model was performed and R^2^ values and p‐values were obtained. Differences were considered statistically significant for p values < 0.05 (visualized as ^*^ *p* < 0.05; ^**^ *p* < 0.01; ^***^ *p* < 0.001; ^****^ *p* < 0.0001). Data is presented as mean ± SD. The amount of samples per statistical analysis are mentioned in the figure legend of each test.

### Ethics Approval

Ethics approval for experiments reported in the submitted manuscript on animal or human subjects was granted. CEA samples were acquired in a manner that fulfilled the declaration of Helsinki and was approved by the hospital's Ethical Research Committee (MEC 2008–147). Iliac crest bone chips were harvested with consent for the use of surgical waste material with a possibility for parental opt‐out and approved by the Medical Ethics Review Committee at the Erasmus MC Medical Ethical Committee (MEC‐2014‐106).

## Conflict of Interest

The authors declare no conflict of interest.

## Supporting information



Supporting Information

## Data Availability

The data that support the findings of this study are available from the corresponding author upon reasonable request.

## References

[1] H. E. Barrett , K. Van der Heiden , E. Farrell , F. J. H. Gijsen , A. C. Akyildiz , J. Biomech. 2019, 87, 1. 30904335 10.1016/j.jbiomech.2019.03.005

[2] W. Herrington , B. Lacey , P. Sherliker , J. Armitage , S. Lewington , Circ. Res. 2016, 118, 535. 26892956 10.1161/CIRCRESAHA.115.307611

[3] P. Libby , J. E. Buring , L. Badimon , G. K. Hansson , J. Deanfield , M. S. Bittencourt , L. Tokgözoğlu , E. F. Lewis , Nat. Rev. Dis. Prim. 2019, 5, 1. 31420554 10.1038/s41572-019-0106-z

[4] A. V. Finn , M. Nakano , J. Narula , F. D. Kolodgie , R. Virmani , Arterioscler. Thromb. Vasc. Biol. 2010, 30, 1282. 20554950 10.1161/ATVBAHA.108.179739

[5] R. Virmani , A. P. Burke , A. Farb , F. D. Kolodgie , J. Am. Coll. Cardiol. 2006, 47, C13. 16631505 10.1016/j.jacc.2005.10.065

[6] C. L. Lendon , M. J. Davies , G. V. R. Born , P. D. Richardson , Atherosclerosis 1991, 87, 87. 1872926 10.1016/0021-9150(91)90235-u

[7] S. E. P. New , E. Aikawa , Arterioscler. Thromb. Vasc. Biol. 2013, 33, 1753. 23766262 10.1161/ATVBAHA.112.300128 PMC3788633

[8] A. J. Moss , A. M. Sim , P. D. Adamson , M. A. Seidman , J. P. M. Andrews , M. K. Doris , A. S. V. Shah , R. BouHaidar , C. J. Alcaide‐Corral , M. C. Williams , J. A. Leipsic , M. R. Dweck , V. E. MacRae , D. E. Newby , A. A. S. Tavares , S. L. Sellers , Sci. Reports 2020, 101, 1. 10.1038/s41598-020-77391-6 PMC7677392 33214599

[9] I. Perrotta , E. Perri , Microsc. Microanal. 2017, 23, 1030. 28874210 10.1017/S1431927617012533

[10] E. Aikawa , M. Nahrendorf , J. L. Figueiredo , F. K. Swirski , T. Shtatland , R. H. Kohler , F. A. Jaffer , M. Aikawa , R. Weissleder , Circulation 2007, 116, 2841. 18040026 10.1161/CIRCULATIONAHA.107.732867

[11] A. Kelly‐Arnold , N. Maldonado , D. Laudier , E. Aikawa , L. Cardoso , S. Weinbaum , Proc. Natl. Acad. Sci. USA 2013, 110, 10741. 23733926 10.1073/pnas.1308814110 PMC3696743

[12] S. H. Rambhia , X. Liang , M. Xenos , Y. Alemu , N. Maldonado , A. Kelly , S. Chakraborti , S. Weinbaum , L. Cardoso , S. Einav , D. Bluestein , Ann. Biomed. Eng. 2012, 40, 1443. 22234864 10.1007/s10439-012-0511-x

[13] N. Maldonado , A. Kelly‐Arnold , D. Laudier , S. Weinbaum , L. Cardoso , Int. J. Cardiovasc. Imaging 2015, 31, 1079. 25837377 10.1007/s10554-015-0650-x PMC5253073

[14] D. Bluestein , Y. Alemu , I. Avrahami , M. Gharib , K. Dumont , J. J. Ricotta , S. Einav , J. Biomech. 2008, 41, 1111. 18258240 10.1016/j.jbiomech.2007.11.029

[15] M. Cilla , D. Monterde , E. Pena , M. A. Martinez , Proc. Inst. Mech. Eng. Part H J. Eng. Med. 2013, 227, 588. 10.1177/0954411913479530 23637269

[16] Y. Vengrenyuk , S. Carlier , S. Xanthos , L. Cardoso , P. Ganatos , R. Virmnani , S. Einav , L. Gilchrist , S. Weinbaum , Proc. Natl. Acad. Sci. USA 2006, 103, 14678. 17003118 10.1073/pnas.0606310103 PMC1595411

[17] J. F. Wenk , P. Papadopoulos , T. I. Zohdi , J. Biomech. Eng. 2010, 132, 091011. 20815645 10.1115/1.4001351

[18] A. Corti , D. Khalil , A. De Paolis , L. Cardoso , J. Mech. Behav. Biomed. Mater. 2023, 141, 105749. 36924613 10.1016/j.jmbbm.2023.105749 PMC10081969

[19] I. Jansen , H. Crielaard , T. Wissing , C. Bouten , F. Gijsen , A. C. Akyildiz , E. Farrell , K. van der Heiden , APL Bioeng. 2023, 7, 036120. 37786532 10.1063/5.0168087 PMC10541963

[20] M. E. Lin , T. M. Chen , M. C. Wallingford , N. B. Nguyen , S. Yamada , C. Sawangmake , J. Zhang , M. Y. Speer , C. M. Giachelli , Cardiovasc. Res. 2016, 112, 606. 27671804 10.1093/cvr/cvw205 PMC5079276

[21] A. Shioi , M. Katagi , Y. Okuno , K. Mori , S. Jono , H. Koyama , Y. Nishizawa , Circ. Res. 2002, 91, 9. 12114316 10.1161/01.res.0000026421.61398.f2

[22] F. Otsuka , K. Sakakura , K. Yahagi , M. Joner , R. Virmani , Arterioscler. Thromb. Vasc. Biol. 2014, 34, 724. 24558104 10.1161/ATVBAHA.113.302642 PMC4095985

[23] R. Kramann , C. Goettsch , J. Wongboonsin , H. Iwata , R. K. Schneider , C. Kuppe , N. Kaesler , M. Chang‐Panesso , F. G. Machado , S. Gratwohl , K. Madhurima , J. D. Hutcheson , S. Jain , E. Aikawa , B. D. Humphreys , Cell Stem Cell. 2016, 19, 628. 27618218 10.1016/j.stem.2016.08.001 PMC5097006

[24] A. P. Sage , Y. Tintut , L. L. Demer , Nat. Rev. Cardiol. 2010, 7, 528. 20664518 10.1038/nrcardio.2010.115 PMC3014092

[25] K. Van der Heiden , A. Hoogendoorn , M. J. Daemen , F. J. H. Gijsen , Thromb. Haemost. 2016, 115, 501. 26607378 10.1160/TH15-07-0614

[26] X. Shi , J. Gao , Q. Lv , H. Cai , F. Wang , R. Ye , X. Liu , Front. Physiol. 2020, 11, 56. 32116766 10.3389/fphys.2020.00056 PMC7013039

[27] E. Ceccherini , A. Cecchettini , I. Gisone , E. Persiani , M. A. Morales , F. Vozzi , Biomedicines 2022, 10, 1. 10.3390/biomedicines10102491 PMC9599098 36289753

[28] A. Shioi , Y. Nishizawa , S. Jono , H. Koyama , M. Hosoi , H. Morii , Arterioscler. Thromb. Vasc. Biol. 1995, 15, 2003. 7583582 10.1161/01.atv.15.11.2003

[29] N. X. Chen , D. Duan , K. D. O'Neill , S. M. Moe , Nephrol. Dial. Transplant. 2006, 21, 3435. 17005530 10.1093/ndt/gfl429

[30] A. Trion , C. Schutte‐Bart , W. H. Bax , J. W. Jukema , A. Laarse , Mol. Cell. Biochem. 2008, 308, 25. 17909945 10.1007/s11010-007-9608-1 PMC2226060

[31] S. Afra , M. M. Matin , Cell Tissue Res. 2020, 380, 1. 31897835 10.1007/s00441-019-03161-0

[32] Z. Gong , L. Niklason , FASEB J. 2008, 22, 1635. 18199698 10.1096/fj.07-087924 PMC2605790

[33] N. F. Huang , S. Li , Regener. Med. 2008, 3, 877.

[34] N. S. Hwang , C. Zhang , Y. S. Hwang , S. Varghese , Wiley Interdiscip. Rev. Syst. Biol. Med. 2009, 1, 97. 20835984 10.1002/wsbm.26

[35] K. Tamama , C. K. Sen , A. Wells , Stem Cells Dev. 2008, 17, 897. 18564029 10.1089/scd.2007.0155 PMC2973839

[36] Y. Shudo , J. E. Cohen , A. B. Goldstone , J. W. MacArthur , J. Patel , B. B. Edwards , M. S. Hopkins , A. N. Steele , L. M. Joubert , S. Miyagawa , Y. Sawa , Y. J. Woo , Cytotherapy 2016, 18, 510. 26971679 10.1016/j.jcyt.2016.01.012 PMC5964977

[37] W. Gu , X. Hong , A. Le Bras , W. N. Nowak , S. I. Bhaloo , J. Deng , Y. Xie , Y. Hu , X. Z. Ruan , Q. Xu , J. Biol. Chem. 2018, 293, 8089. 29643181 10.1074/jbc.RA118.001739 PMC5971462

[38] J. Brun , K. A. Lutz , K. M. H. Neumayer , G. Klein , T. Seeger , T. Uynuk‐Ool , K. Wörgötter , S. Schmid , U. Kraushaar , E. Guenther , B. Rolauffs , W. K. Aicher , M. L. Hart , PLoS One 2015, 10, 1. 10.1371/journal.pone.0145153 PMC4684225 26673782

[39] S. Sivaraman , J. Hedrick , S. Ismail , C. Slavin , R. R. Rao , Int. J. Mol. Sci. 2021, 22, 10335. 34638675 10.3390/ijms221910335 PMC8508589

[40] W. S. Park , S. C. Heo , E. S. Jeon , D. H. Hong , Y. K. Son , J. H. Ko , H. K. Kim , S. Y. Lee , J. H. Kim , J. Han , Am. J. Physiol. – Cell Physiol. 2013, 305, C377. 23761629 10.1152/ajpcell.00404.2012 PMC3891216

[41] N. Kobayashi , T. Yasu , H. Ueba , M. Sata , S. Hashimoto , M. Kuroki , M. Saito , M. Kawakami , Exp. Hematol. 2004, 32, 1238. 15588948 10.1016/j.exphem.2004.08.011

[42] S. Ghazanfari , M. Tafazzoli‐Shadpour , M. A. Shokrgozar , Biochem. Biophys. Res. Commun. 2009, 388, 601. 19695226 10.1016/j.bbrc.2009.08.072

[43] R. Park , J. W. Yoon , J. H. Lee , S. W. Hong , J. H. Kim , J. Nanobiotechnol. 2022, 20, 1. 10.1186/s12951-021-01225-4 PMC8725258 34983551

[44] S. Liu , J. Ren , Y. Hu , F. Zhou , L. Zhang , Cell Regen. 2024, 13, 1. 39604763 10.1186/s13619-024-00207-9 PMC11602941

[45] S. Tsai , S. T. Hollenbeck , E. J. Ryer , R. Edlin , D. Yamanouchi , R. Kundi , C. Wang , B. Liu , K. C. Kent , Am. J. Physiol. – Hear. Circ. Physiol. 2009, 297, H540. 10.1152/ajpheart.91478.2007 PMC2724222 19525370

[46] X. Guo , World J. Biol. Chem. 2012, 3, 41. 22451850 10.4331/wjbc.v3.i3.41 PMC3312200

[47] S. A. Steitz , M. Y. Speer , G. Curinga , H. Y. Yang , P. Haynes , R. Aebersold , T. Schinke , G. Karsenty , C. M. Giachelli , Circ. Res. 2001, 89, 1147. 11739279 10.1161/hh2401.101070

[48] J.‐S. Shao , S.‐L. Cheng , J. M. Pingsterhaus , N. Charlton‐Kachigian , A. P. Loewy , D. A. Towler , J. Clin. Invest. 2005, 115, 1210. 15841209 10.1172/JCI24140 PMC1077175

[49] M. C. Andrade , L. S. Carmo , E. Farias‐Silva , M. Liberman , Atherosclerosis 2017, 265, 14. 28829997 10.1016/j.atherosclerosis.2017.07.028

[50] C. H. Byon , Y. Sun , J. Chen , K. Yuan , X. Mao , J. M. Heath , P. G. Anderson , Y. Tintut , L. L. Demer , D. Wang , Y. Chen , Arterioscler. Thromb. Vasc. Biol. 2011, 31, 1387. 21454810 10.1161/ATVBAHA.110.222547 PMC3098301

[51] T. M. Liu , E. H. Lee , Tissue Eng. – Part B Rev. 2013, 19, 254. 23150948 10.1089/ten.teb.2012.0527 PMC3627420

[52] C. Vater , P. Kasten , M. Stiehler , Acta Biomater. 2011, 7, 463. 20688199 10.1016/j.actbio.2010.07.037

[53] J. J. Patel , L. E. Bourne , B. K. Davies , T. R. Arnett , V. E. MacRae , C. P. Wheeler‐Jones , I. R. Orriss , Exp. Cell Res. 2019, 380, 100. 31004580 10.1016/j.yexcr.2019.04.020 PMC6520648

[54] J. D. Hutcheson , C. Goettsch , S. Bertazzo , N. Maldonado , J. L. Ruiz , W. Goh , K. Yabusaki , T. Faits , C. Bouten , G. Franck , T. Quillard , P. Libby , M. Aikawa , S. Weinbaum , E. Aikawa , Nat. Mater. 2016, 15, 335. 26752654 10.1038/nmat4519 PMC4767675

[55] E. Maeda , K. Ichikawa , K. Murase , K. Nagayama , T. Matsumoto , J. Biomech. 2018, 78, 94. 30060920 10.1016/j.jbiomech.2018.07.024

[56] H. N. Hutson , T. Marohl , M. Anderson , K. Eliceiri , P. Campagnola , K. S. Masters , PLoS One 2016, 11, 1. 10.1371/journal.pone.0163858 PMC5042542 27685946

[57] Y. J. Seong , I. G. Kang , E. H. Song , H. E. Kim , S. H. Jeong , Adv. Healthcare Mater. 2017, 6, 1700966. 10.1002/adhm.201700966 29076295

[58] X. Q. Zheng , J. Huang , J. Lin , C. L. Song , J. Adv. Res. 2023, 49, 63. 36115662 10.1016/j.jare.2022.08.019 PMC10334135

[59] K. Balachandran , P. Sucosky , H. Jo , A. P. Yoganathan , Am. J. Pathol. 2010, 177, 49. 20489151 10.2353/ajpath.2010.090631 PMC2893650

[60] A. J. Janvier , E. G. Pendleton , L. J. Mortensen , D. C. Green , J. R. Henstock , E. G. Canty‐Laird , J. Tissue Eng. 2022, 11, 1. 10.1177/20417314221130486 PMC9629721 36339372

[61] J. Tyson , K. Bundy , C. Roach , H. Douglas , V. Ventura , M. F. Segars , O. Schwartz , C. L. Simpson , Bioengineering 2020, 7, 1. 10.3390/bioengineering7030088 PMC7552614 32781528

[62] F. Wernig , M. Mayr , Q. Xu , Hypertension 2003, 41, 903. 12642506 10.1161/01.HYP.0000062882.42265.88

[63] N. Maldonado , A. Kelly‐Arnold , D. Laudier , S. Weinbaum , L. Cardoso , Int. J. Cardiovasc. Imaging 2015, 315, 1079. 10.1007/s10554-015-0650-x PMC5253073 25837377

[64] L. Cardoso , A. Kelly‐Arnold , N. Maldonado , D. Laudier , S. Weinbaum , J. Biomech. 2014, 47, 870. 24503048 10.1016/j.jbiomech.2014.01.010 PMC4019736

[65] I. Jansen , H. Crielaard , T. Wissing , C. Bouten , F. Gijsen , A. C. Akyildiz , E. Farrell , K. van der Heiden , APL Bioeng. 2023, 7, 36120. 10.1063/5.0168087 PMC10541963 37786532

[66] L. J. Smith , A. C. Deymier , J. J. Boyle , Z. Li , S. W. Linderman , J. D. Pasteris , Y. Xia , G. M. Genin , S. Thomopoulos , Interface Focus 2016, 6, 20150070. 26855755 10.1098/rsfs.2015.0070 PMC4686244

[67] S. Dabrowska , M. Ekiert‐Radecka , J. Karbowniczek , W. P. Weglarz , M. Heljak , M. Lojkowski , R. Obuchowicz , W. Swieszkowski , A. Mlyniec , Acta Biomater. 2023, 166, 360. 37172636 10.1016/j.actbio.2023.05.010

[68] A. Irkle , A. T. Vesey , D. Y. Lewis , J. N. Skepper , J. L. E. Bird , M. R. Dweck , F. R. Joshi , F. A. Gallagher , E. A. Warburton , M. R. Bennett , K. M. Brindle , D. E. Newby , J. H. Rudd , A. P. Davenport , Nat. Commun. 2015, 6, 7495. 26151378 10.1038/ncomms8495 PMC4506997

[69] M. Burgmaier , A. Milzi , R. Dettori , K. Burgmaier , N. Marx , S. Reith , PLoS One 2018, 13, 0205984. 10.1371/journal.pone.0205984 PMC6200236 30356326

[70] A. Corti , D. Khalil , A. De Paolis , L. Cardoso , J. Mech. Behav. Biomed. Mater. 2023, 141, 105749. 36924613 10.1016/j.jmbbm.2023.105749 PMC10081969

[71] K.‐A. Maldonado , C. Weinbaum , J. Biomech. 2013, 46, 396. 23218838 10.1016/j.jbiomech.2012.10.040 PMC4019735

[72] A. Corti , A. De Paolis , P. Grossman , P. A. Dinh , E. Aikawa , S. Weinbaum , L. Cardoso , Front. Cardiovasc. Med. 2022, 9, 1. 10.3389/fcvm.2022.1019917 PMC9583261 36277774

[73] R. D. Johnston , R. T. Gaul , C. Lally , Acta Biomater. 2021, 124, 291. 33571712 10.1016/j.actbio.2021.02.008

[74] J. G. Snedeker , A. Gautieri , Muscles. Ligaments Tendons J. 2014, 4, 303. 25489547 PMC4241420

[75] M. J. Barnes , R. W. Farndale , Exp. Gerontol. 1999, 34, 513. 10817807 10.1016/s0531-5565(99)00038-8

[76] I. Jansen , R. Cahalane , R. Hengst , A. Akyildiz , E. Farrell , F. Gijsen , E. Aikawa , K. van der Heiden , T. Wissing , Basic Res. Cardiol. 2024, 119, 193. 38329498 10.1007/s00395-024-01033-5 PMC11008085

[77] Y. F. Yasenchuk , E. S. Marchenko , S. V. Gunter , G. A. Baigonakova , O. V. Kokorev , A. A. Volinsky , E. B. Topolnitsky , Mater 2021, 14, 6256. 10.3390/ma14216256 PMC8585136 34771782

[78] P. K. Paritala , P. K. D. V. Yarlagadda , R. Kansky , J. Wang , J. B. Mendieta , Y. T. Gu , T. McGahan , T. Lloyd , Z. Li , Front. Bioeng. Biotechnol. 2020, 8, 500870. 10.3389/fbioe.2020.00060 PMC7026010 32117939

[79] D. Balzani , Encycl. Contin. Mech. 2018, 1.

[80] E. Peña , J. A. Peña , M. Doblaré , Int. J. Solids Struct. 2009, 46, 1727.

[81] K. Dey , S. Agnelli , E. Borsani , L. Sartore , Gels 2021, 7, 277. 34940337 10.3390/gels7040277 PMC8701964

[82] E. Aikawa , M. C. Blaser , Arterioscler. Thromb. Vasc. Biol. 2021, 41, 117. 33115271 10.1161/ATVBAHA.120.314704 PMC7832175

[83] J. Brun , K. A. Lutz , K. M. H. Neumayer , G. Klein , T. Seeger , T. Uynuk‐Ool , K. Wörgötter , S. Schmid , U. Kraushaar , E. Guenther , B. Rolauffs , W. K. Aicher , M. L. Hart , PLoS One 2015, 10, 0145153. 10.1371/journal.pone.0145153 PMC4684225 26673782

[84] T. B. Wissing , K. Van der Heiden , S. M. Serra , A. I. P. M. Smits , C. V. C. Bouten , F. J. H. Gijsen , Sci. Rep. 2022, 12, 1. 35361847 10.1038/s41598-022-08425-4 PMC8971478

[85] N. De Jonge , F. M. W. Kanters , F. P. T. Baaijens , C. V. C. Bouten , Ann. Biomed. Eng. 2013, 41, 763. 23184346 10.1007/s10439-012-0704-3

[86] G. Huszar , J. Maiocco , F. Naftolin , Anal. Biochem. 1980, 105, 424. 7457846 10.1016/0003-2697(80)90481-9

[87] E. E. Van Haaften , T. B. Wissing , M. C. M. Rutten , J. A. Bulsink , K. Gashi , M. A. J. Van Kelle , A. I. P. M. Smits , C. V. C. Bouten , N. A. Kurniawan , Tissue Eng. – Part C Methods 2018, 24, 418. 29877143 10.1089/ten.TEC.2018.0104

[88] H. Crielaard , S. G. Torun , T. B. Wissing , P. Muñoz , M. de , G. J. Kremers , F. J. H. Gijsen , K. Van Der Heiden , A. C. Akyildiz , J. Vis. Exp. 2022, 189, e64334. 10.3791/64334 36440849

[89] S. G. Torun , P. Munoz , M. de , H. Crielaard , H. J. M. Verhagen , G. J. Kremers , A. F. W. van der Steen , A. C. Akyildiz , Acta Biomater. 2023, 164, 293. 37086826 10.1016/j.actbio.2023.04.022

[90] A. Paini , P. Boutouyrie , D. Calvet , M. Zidi , E. Agabiti‐Rosei , S. Laurent , Stroke 2007, 38, 117. 17158335 10.1161/01.STR.0000251796.38954.b2

[91] J. Huang , S. Tu , C. Li , H. Hong , Z. Wang , L. Chen , J. L. Gutiérrez‐Chico , W. Wijns , J. Soc. Cardiovasc. Angiogr. Interv. 2022, 0, 100570. 10.1016/j.jscai.2022.100570 PMC11307920 39129795

[92] M. Stritt , A. K. Stalder , E. Vezzali , PLoS Comput. Biol. 2020, 16, 1007313. 10.1371/journal.pcbi.1007313 PMC7028292 32023239

