# Supplementary material for: Modelling Atherosclerotic Plaque Cap Mechanics: Microcalcifications Reduce Mechanical Properties in Mesenchymal Stromal Cell‐Based Model
Source: Adv Biol (Weinh). 2025 Jul 9;9(10):e00106. doi: 10.1002/adbi.202500106 (PMC12517318; doi:10.1002/adbi.202500106)
Supplement: Supplementary file 1 — Supporting Information [file ADBI-9-e00106-s001.docx]

**Supplementary information to:** **Modelling atherosclerotic plaque cap mechanics: microcalcifications reduce mechanical properties in mesenchymal stromal cell-based model.**

Authors: Imke L. Jansen^1^, Deniz Sahin^3^, Frank J.H. Gijsen^1,2^, Eric Farrell^3^*, Kim van der Heiden^1^*

*both authors contributed equally,

Corresponding author Eric Farrell [e.farrell@erasmusmc.nl](mailto:e.farrell@erasmusmc.nl)

1. Department of Biomedical Engineering, Thorax Center Erasmus MC, University Medical Center Rotterdam, Rotterdam, The Netherlands
2. Department of Biomechanical Engineering, Delft University of Technology, Delft, The Netherlands
3. Department of Oral and Maxillofacial Surgery, Erasmus MC, University Medical Center Rotterdam, Rotterdam, The Netherlands

Supp. Table 1: Overview of donors and for which analysis the samples were used. Only donor 3-4-5 were cultured until week 6 and used for subsequent analysis.

**
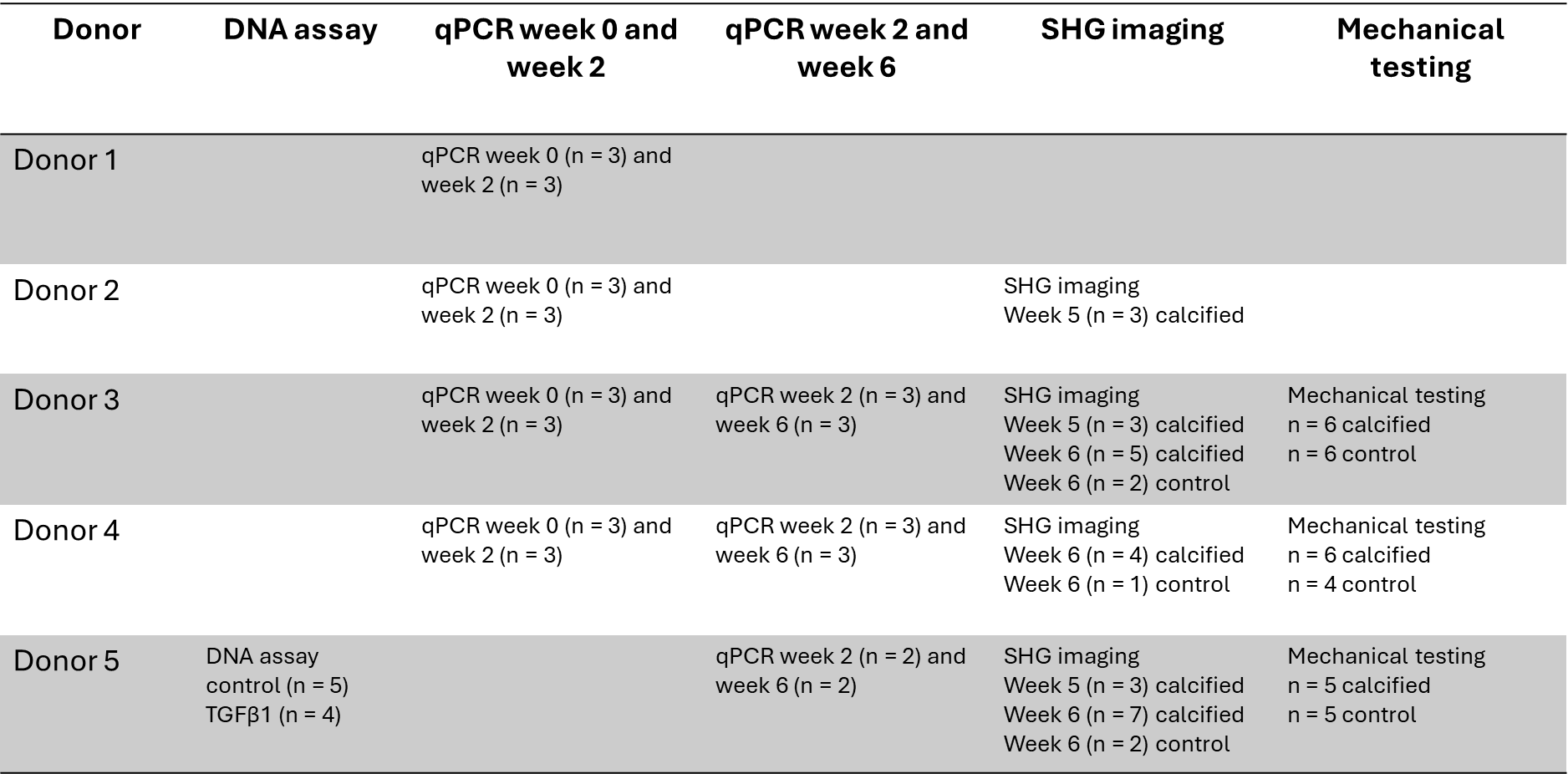
**


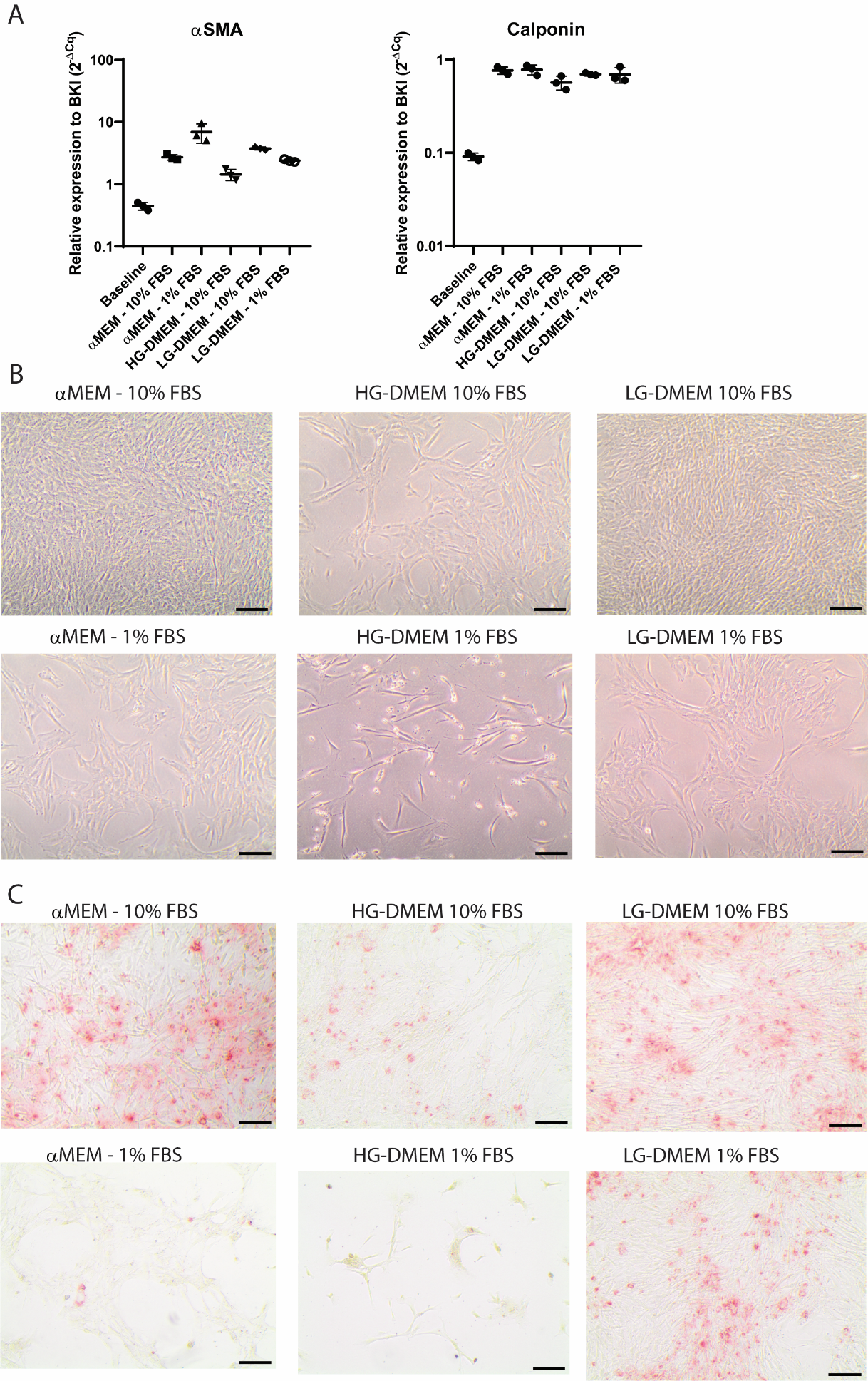


Supp. Figure 1 Monolayer differentiation and calcification (A) qPCR data of αSMA and Calponin in monolayer experiment (n =3). (B) Brightfield images of cells after 6 days of differentiation in various media types. (C) Alizarin red staining after total of 21 days of culture. (B-C) Scale = 200 µm


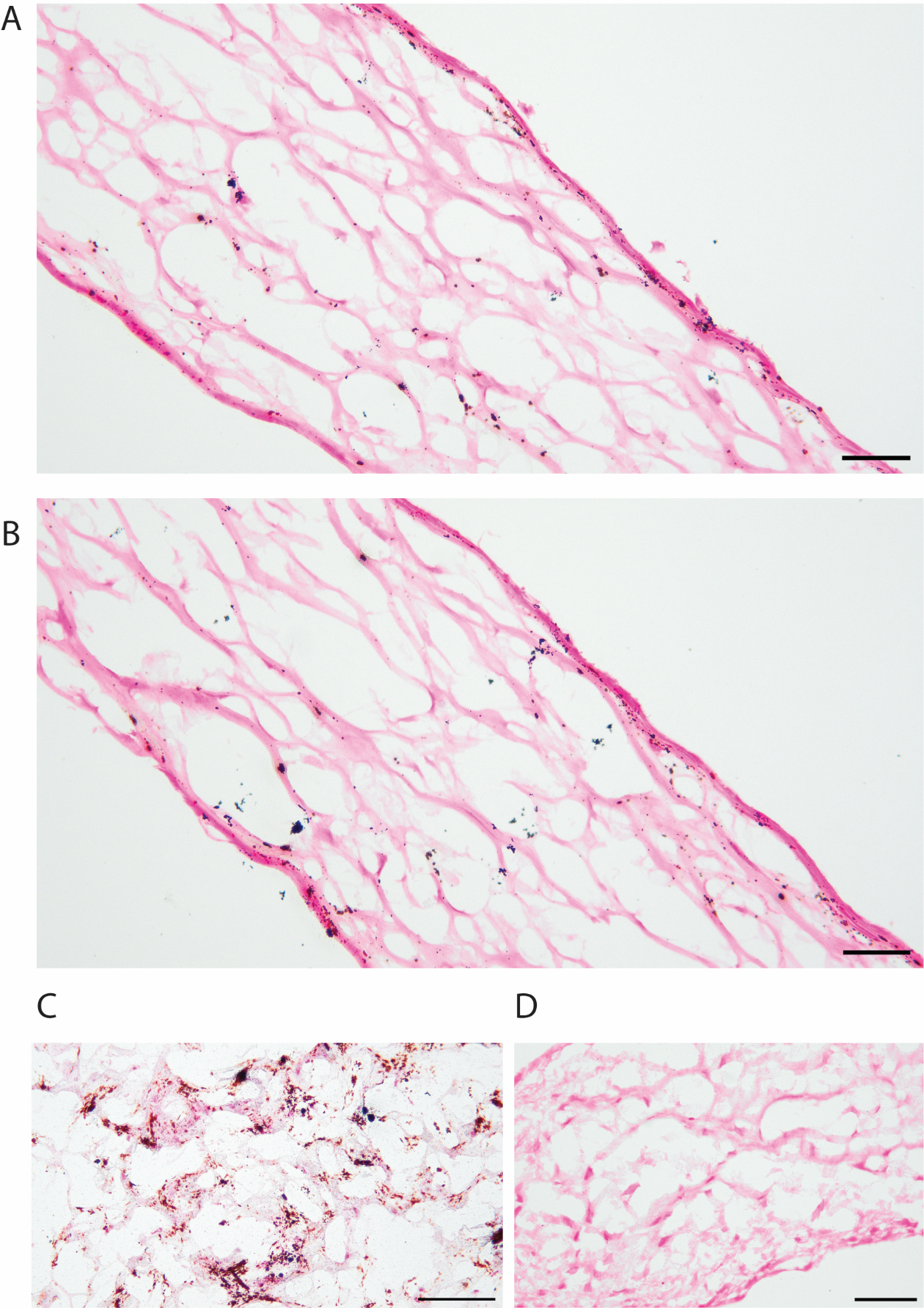


Supp. Figure 2 Von Kossa staining after 6 weeks of culture (A-B) Microcalcifications are visible through the entire depth of the samples. (C) Microcalcifications follow the matrix fibres (D) Control sample showing no microcalcification formation. Scale: (A-B) 50 µm (C-D) 25 µm

|  | **Donor 3** | **Donor 4** | **Donor 5** |
| --- | --- | --- | --- |
| **H&E** | **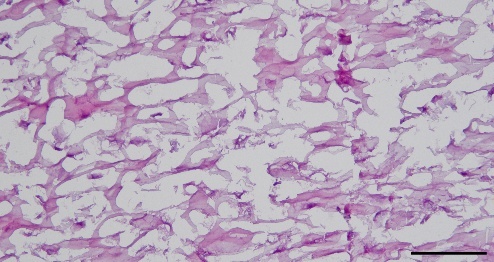** | **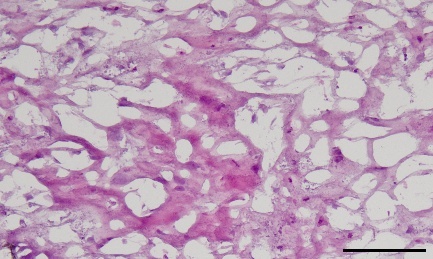** | 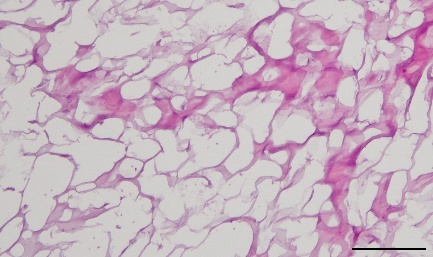 |
| **Picrosirius Red** | 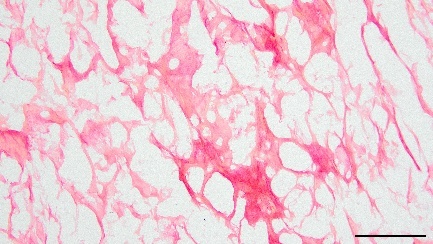 | 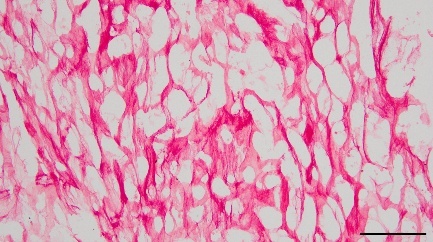 | 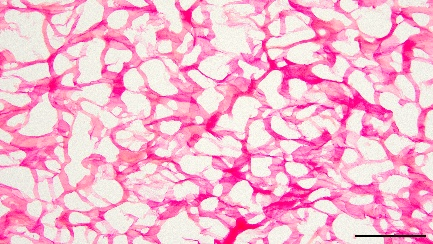 |
| **Von Kossa** | 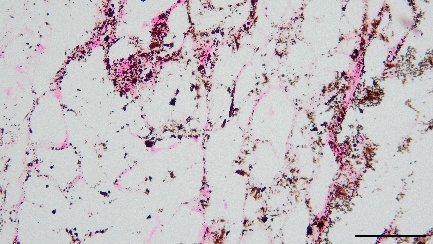 | 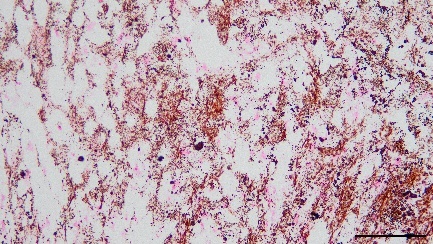 | 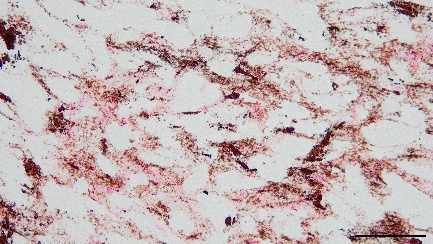 |

Supp. Fig. 3: Representative images of the 3 donors cultured until week 6. H&E staining, Picrosirius Red staining for collagen and Von Kossa staining for calcifications are shown. Scale = 100 μm. Donor 1 and 2 are not shown. Donor 1 was used for qPCR analysis at baseline and day 14. These tissues were not cultured until the 6-week endpoint, so no representative histological image can be given. Donor 2 was used for qPCR analysis at baseline and at day 14. 3 samples were cultured until week 5 for SHG imaging and afterwards harvested for qPCR analysis at week 5 (data not shown in paper).


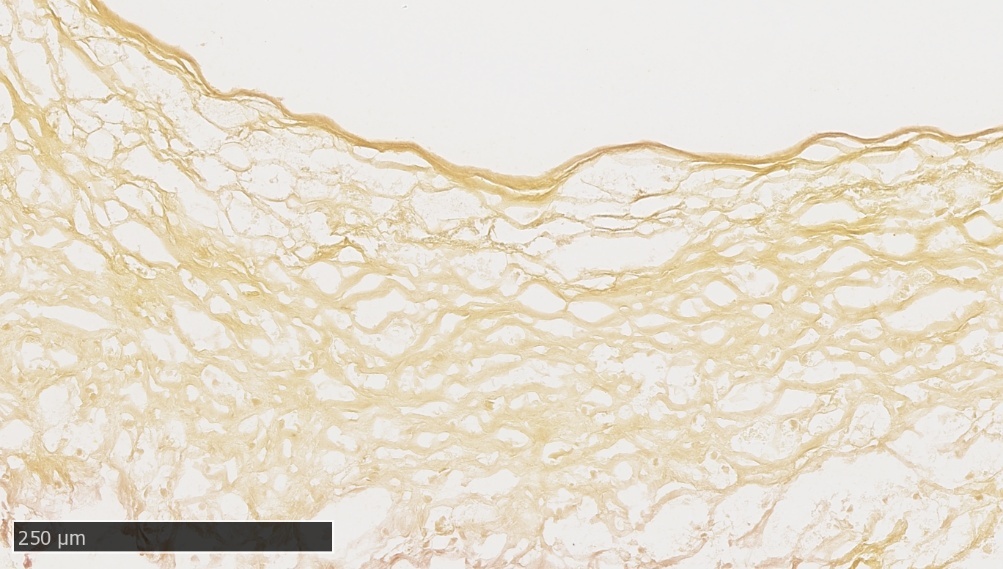


Supp. Fig. 4: Representative image of human fibrous cap obtained from carotid endarterectomy sample, showing the matrix of the cap with fibers and voids in between.

Supp. Fig. 5: qPCR of osteogenic transcription factors RUNX2 and Msx2 at week 2 and 6 of culture (3 donors). Mixed effects model with Bonferroni’s multiple comparisons showed no significant differences.
